# Tuning Butyrylcholinesterase Inactivation and Reactivation by Polymer‐Based Protein Engineering

**DOI:** 10.1002/advs.201901904

**Published:** 2019-11-13

**Authors:** Libin Zhang, Stefanie L. Baker, Hironobu Murata, Nicholas Harris, Weihang Ji, Gabriel Amitai, Krzysztof Matyjaszewski, Alan J. Russell

**Affiliations:** ^1^ Center for Polymer‐Based Protein Engineering Carnegie Mellon University 5000 Forbes Avenue Pittsburgh PA 15213 USA; ^2^ Department of Biomedical Engineering Carnegie Mellon University 5000 Forbes Avenue Pittsburgh PA 15213 USA; ^3^ Department of Biotechnology Engineering ORT Braude Academic College Karmiel POB78 Israel; ^4^ Wohl Drug Discovery Institute Nancy and Stephen Grand Israel National Center for Personalized Medicine (G‐INCPM) Weizmann Institute of Science Rehovot 760001 Israel; ^5^ Department of Chemistry Department of Chemical Engineering Carnegie Mellon University 4400 Fifth Avenue Pittsburgh PA 15213 USA

**Keywords:** atom transfer radical polymerization, butyrylcholinesterase, organophosphate nerve agents, oximes, protein–polymer conjugates

## Abstract

Organophosphate nerve agents rapidly inhibit cholinesterases thereby destroying the ability to sustain life. Strong nucleophiles, such as oximes, have been used as therapeutic reactivators of cholinesterase‐organophosphate complexes, but suffer from short half‐lives and limited efficacy across the broad spectrum of organophosphate nerve agents. Cholinesterases have been used as long‐lived therapeutic bioscavengers for unreacted organophosphates with limited success because they react with organophosphate nerve agents with one‐to‐one stoichiometries. The chemical power of nucleophilic reactivators is coupled to long‐lived bioscavengers by designing and synthesizing cholinesterase‐polymer‐oxime conjugates using atom transfer radical polymerization and azide‐alkyne “click” chemistry. Detailed kinetic studies show that butyrylcholinesterase‐polymer‐oxime activity is dependent on the electrostatic properties of the polymers and the amount of oxime within the conjugate. The covalent coupling of oxime‐containing polymers to the surface of butyrylcholinesterase slows the rate of inactivation of paraoxon, a model nerve agent. Furthermore, when the enzyme is covalently inhibited by paraoxon, the covalently attached oxime induced inter‐ and intramolecular reactivation. Intramolecular reactivation will open the door to the generation of a new class of nerve agent scavengers that couple the speed and selectivity of biology to the ruggedness and simplicity of synthetic chemicals.

## Graphical abstract

Butyrylcholinesterase (BChE)‐polymer‐oxime conjugates are synthesized by in situ atom transfer radical polymerization and azide‐alkyne “click” chemistry. The conjugate's activity depends on the electrostatic properties of the polymers and the oxime to BChE ratio. The oximes on the surface of BChE delay irreversible inhibition by paraoxon, and furthermore, are able to reactivate inhibited BChE through intra‐ and intermolecular interactions.

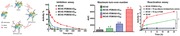

## 1 Introduction

Organophosphate nerve agents (OPNAs) are potent inhibitors of the essential components of cholinergic neurotransmission, cholinesterases (ChEs).^1^ The inhibition is irreversible and results from the formation of a covalent bond between the OPNA and the serine residue at the enzyme active site. ChE inhibition causes excessive accumulation of neurotransmitters in cholinergic synapses and leads to life‐threatening toxic manifestations including loss of consciousness, breathing failure, paralysis, and eventually death.^2^, ^3^, ^4^ OPNAs inactivate serum cholinesterase (butyrylcholinesterase (BChE)) and can also cross the blood brain barrier, accumulating in the central nervous system where they inactivate acetylcholinesterase (AChE) at synaptic junctions before slowly partitioning back into the circulation.^5^ OPNAs have been used in terrorism, such as the nerve gas attack at a Tokyo subway in 1995, and in state‐sponsored civilian attacks such as the recent devastating attacks on Syrian citizens.^6^, ^7^, ^8^, ^9^, ^10^, ^11^ Today, there are no truly effective therapies or broadly applicable OPNA detoxifiers.

The seminal studies on reactivation of covalently‐inhibited ChEs^12^ indicated that enzyme‐bound OPNAs could be released by nucleophiles that target the phosphoryl bond with the active site serine, thereby restoring activity. In vivo, strong nucleophiles, such as oximes, rapidly displace the organophosphate (OP) moiety from the active site and may also partially deactivate slowly circulating OPNAs.^13^, ^14^, ^15^ During the past several decades, large numbers of oximes have been developed as reactivators for OPNA‐inhibited ChEs.^16^, ^17^, ^18^, ^19^, ^20^ Several pyridinium aldoximes, including 2‐PAM, bispyridinium oxime HI‐6, and obidoxime, are currently approved antidotes for the treatment of acute OPNA poisoning when combined with atropine and benzodiazepine.^21^, ^22^ Numerous efforts have been made to develop an array of second generation nucleophilic reactivators, such as imidazolium, pyridinium–imidazolium, and quinuclidinium–imidazolium compounds, that have a variety of heterocyclic structures.^5^, ^23^, ^24^, ^25^, ^26^, ^27^ However, even next generation oxime therapies require parenteral administration and are limited by short lifetimes in circulation and by their inability to traverse the blood brain barrier.

BChE hydrolyzes numerous esters and, in vivo, can act stoichiometrically as a first line of defense against OPNA poisoning. The active site of human, monomeric BChE is located at the bottom of a 20 Å deep and narrow active site gorge.^28^, ^29^, ^30^ A BChE tetramer model has shown that four BChE monomers interact via a four‐helix bundle at the tetramerization domain.^31^ A polyproline‐rich peptide inserts into the center of the four‐helix bundle to provide tetrameric form. The enzyme is relatively stable in solution, making it an attractive target for protein atom‐transfer radical polymerization (ATRP).^32^ Highly purified human BChE has been intravenously injected to scavenge OPNAs and was proven effective before and after OPNA exposure.^33^, ^34^, ^35^, ^36^, ^37^ However, using human BChE as a sole OPNA bioscavenger has limitations. First, the reaction between BChE and OPNA is stoichiometric. One macromolecule of BChE only detoxifies one molecule of OPNA in an irreversible process. Second, the molecular weight (*M*_w_) of BChE monomer is 85 kDa, which is 500–600 times higher than OPNAs such as sarin (140 Da) or VX (267 Da), so effective bioscavenging requires a very large amount of BChE (100–500 mg per patient).^38^

We have had a long‐term interest in the use of biology to protect against OPNAs^39^ and we therefore decided to explore whether ChEs could be modified with oxime‐containing polymers that would concentrate potentially intramolecular reactivating nucleophiles around the enzyme active site. Biologically active polymer‐oximes would also have the potential to be long‐lived direct OPNA detoxifiers. We focused on human plasma BChE as our model enzyme.^40^ In addition to plasma, many organs and tissues such as the liver, skin, striated muscle, smooth muscle, lung, and brain contain BChE.^32^ BChE has a half‐life of 12 d in human blood circulation with average concentrations from 3.5 to 9.3 mg L^−1^.^41^, ^42^, ^43^, ^44^, ^45^, ^46^

Attachment of synthetic polymers to form protein–polymer conjugates combines the properties of both biologic and synthetic materials with improved properties in a complex biological environment.^47^, ^48^, ^49^ For example, attaching poly(ethylene glycol) (PEG) to a protein (PEGylation) increases solubility and stability, extends blood circulation time, and reduces potential immunogenicity.^50^, ^51^ A new approach to making protein–polymer conjugates, “grafting‐from,” places polymerization initiators on the surface of a protein, followed by in situ growth of polymer from the initiation points.^52^, ^53^, ^54^, ^55^, ^56^ Compared to the “grafting‐to” method,^57^, ^58^, ^59^, ^60^ “grafting‐from” leads to high yields of protein–polymer conjugates and significantly less complex purification. ATRP, which was developed in the early 1990s, has proven to be a powerful technique to control polymer chain length, producing a narrow size distribution and a large library of available architectures.^61^, ^62^, ^63^, ^64^ Recently, we have generated a series of protein–polymer conjugates, using ATRP, with increased stability at low pH (pH 1), high temperature, organic solvent solubility, and transepithelial protein transport.^65^, ^66^, ^67^, ^68^, ^69^ The opportunity now exists to generate polymer–protein conjugates that contain multiple copies of reactivating nucleophiles on BChE, thereby concentrating the nucleophile to an extent that the BChE protein cores become potential decontaminating enzymes for OPNAs.

**Figure 1:**
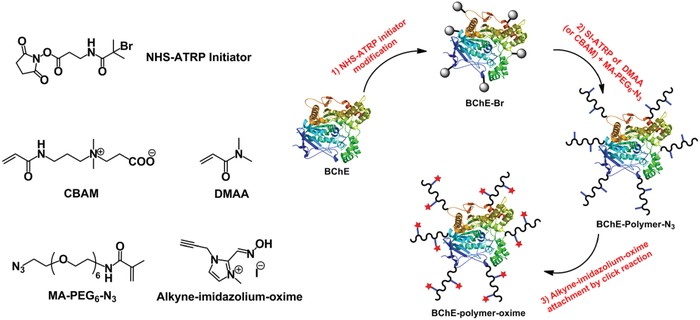
The use of atom‐transfer radical polymerization (ATRP) and click chemistry to synthesize butyrylcholinesterase‐polymer‐oxime conjugates. Additional acronyms: *N*‐hydroxysuccinimide (NHS), carboxybetaine acrylamide (CBAM), *N*,*N*‐dimethylacrylamide (DMAA), *N*‐(20‐azido‐3,6,9,12,15,18‐hexaoxaicosyl)methacrylamide (MA‐PEG_6_‐N_3_), butyrylcholinesterase (BChE).

Herein, we describe the use of protein‐ATRP to synthesize BChE‐polymer‐oxime conjugates for use in surface decontamination of OPNAs. The conjugates were prepared by “grafting‐from” in three steps. First, ATRP initiators were attached to lysine residues located on the surface of BChE. Next, random azide copolymers were grown in situ from the BChE macroinitiator using ATRP. Finally, an alkyne‐imidazolium‐oxime was attached to the polymers by azide‐alkyne “click” chemistry (**Figure** **1**). We hypothesized that covalently linked oximes on each polymer chain in the protein–polymer conjugate would significantly increase the local nucleophile concentration and possibly enable intramolecular protection and/or reactivation. This approach would essentially convert the biological target of OPNAs (ChEs) into an OPNA detoxifying agent without a limitation of one‐to‐one stoichiometry.

## 2 Results and Discussion

### 2.1 Synthesis of BChE‐Polymer‐Oxime Conjugates

BChE‐polymer‐oxime conjugates were prepared using “grafting‐from” polymer‐based protein engineering and alkyne‐azide cycloaddition “click” chemistry.^70^ Prior to conjugate synthesis, an amino‐reactive ATRP initiator (NHS‐Br) was covalently reacted with BChE to create a BChE biomacroinitiator. A ninhydrin assay was used to determine that, on average, 6.5 initiators were attached per BChE monomer under the conditions used for protein modification.^71^ Thus, in all the studies described in detail below, each tetramer contained an average of 26 initiators from which polymer chains could grow. The comonomers and number of oximes per polymer chains were varied while the total number of polymer chains were held constant, as grafting density was previously shown to influence enzymatic activity.^72^, ^73^, ^74^

**Figure 2:**
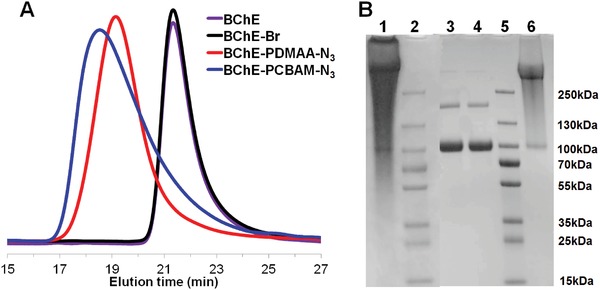
Characterization of BChE, BChE‐Br, and BChE‐Polymer conjugates. A) GPC analysis of BChE, BChE‐Br, BChE‐PDMAA‐N_3_, and BChE‐PCBAM‐N_3_ conjugates. B) SDS‐PAGE analysis of BChE, BChE‐Br, BChE‐PDMAA‐N_3_, and BChE‐PCBAM‐N_3_ conjugates. Lane 1: BChE‐PCBAM‐N_3_; Lane 2: Marker; Lane 3: BChE; Lane 4: BChE‐Br; Lane 5: Marker; Lane 6: BChE‐PDMAA‐N_3_.

Azide‐containing BChE‐polymer conjugates were synthesized using ATRP from the bio‐macroinitiator (Figure 1). In situ ATRP of neutral poly(*N*,*N*‐dimethylacrylamide) (PDMAA) or zwitterionic poly (carboxybetaine acrylamide) (PCBAM) was performed in the presence of an azide comonomer (MA‐PEG_6_‐N_3_) which generated BChE‐PDMAA‐N_3_ and BChE‐PCBAM‐N_3_ conjugates. After purification by ultrafiltration with a 100 kDa cut‐off membrane, the conjugates were characterized by gel permeation chromatography (GPC). GPC analyses of BChE, BChE‐Br, and BChE‐polymer‐azide conjugates showed that the BChE and BChE‐Br peaks at 21.3 min almost disappeared after the in situ copolymerization, accompanied by the emergence of new peaks at 19.2 and 18.6 min for BChE‐PDMAA‐N_3_ and BChE‐PCBAM‐N_3_, respectively indicating polymer growth (**Figure** **2**A). Sodium dodecyl sulfate polyacrylamide gel electrophoresis (SDS‐PAGE) analyses showed that the bands corresponding to BChE and BChE‐Br (≈85 kDa for monomer and ≈170 kDa for dimer) almost disappeared, accompanied by the appearance of new broad bands with higher molecular weights (>300 kDa) corresponding to the BChE‐PDMAA‐N_3_ and BChE‐PCBAM‐N_3_ conjugates (Figure 2B). The hydrodynamic diameters of the protein starting materials (*D*_h_) also increased after protein‐ATRP. The intensity *D*_h_'s of native BChE and BChE‐Br enzymes were 16.5 ± 5.8 and 16.4 ± 5.9 nm, respectively. The BChE‐PDMAA‐N_3_ and BChE‐PCBAM‐N_3_ conjugates grew in size to 50.4 ± 22.3 and 88.4 ± 51.1 nm, respectively (Figure S1, Supporting Information). Polymerization was further confirmed using ^1^H NMR to determine the ratios of azide monomers to DMAA (1:12.2) or CBAM (1:18.5) on the polymer backbone (Figure S2, Supporting Information). The covalently attached polymers of BChE‐PCBAM‐N_3_ were cleaved by acid hydrolysis and characterized by GPC to determine polymer molecular weight and polydispersity index (*Ð*). The weight average molecular weight of cleaved PCBAM‐N_3_ was 95.3 kDa (*Ð* ≈ 2.0) (Figure S3, Supporting Information). The polymers from BChE‐PDMAA‐N_3_ were cleaved by Proteinase K induced digestion.^56^ SDS‐PAGE confirmed the successful cleavage of PDMAA‐N_3_ from the conjugate (Figure S3, Supporting Information). The weight average molecular weight of cleaved PDMAA‐N_3_ was 87.8 kDa (*Ð* ≈ 1.7) (**Table** **1**).

**Table 1:** Characterization of BChE‐polymer conjugates

	Conjugate *M*_w_ [kDa]	Cleaved polymer (3‐Column GPC)		Oximes/BChE monomer
		*M*_w_ [kDa]	*Ð* [*M*_w_/*M*_n_]	
BChE‐PDMAA‐N_3_	656	87.8	1.7	–
BChE‐PCBAM‐N_3_	704	95.3	2.0	–
BChE‐PDMAA‐IO_15_	660	88.5	1.7	15
BChE‐PDMAA‐IO_44_	669	89.8	1.7	44
BChE‐PDMAA‐IO_69_	676	90.9	1.7	69
BChE‐PCBAM‐IO_8_	706	95.6	2.0	8
BChE‐PCBAM‐IO_49_	718	97.5	2.0	49
BChE‐PCBAM‐IO_90_	730	99.3	2.0	90

Alkyne‐imidazolium‐oxime was then coupled to the azido side chains of BChE‐PDMAA‐N_3_ and BChE‐PCBAM‐N_3_ at different ratios using CuSO_4_/NaAsc‐catalyzed azide‐alkyne cycloaddition “click” chemistry.^75^ The number of oximes per BChE monomer was varied by modifying the stoichiometry of alkyne‐imidazolium‐oxime to BChE‐PDMAA‐N_3_ or BChE‐PCBAM‐N_3_. Either 20‐fold, 60‐fold, or 120‐fold of oximes was used to modify BChE‐PDMAA‐N_3_ and BChE‐PCBAM‐N_3_ to prepare BChE‐PDMAA‐IO and BChE‐PCBAM‐IO conjugates. Azide group conversions were 4%, 13%, and 20% with increasing stoichiometry for BChE‐PDMAA‐IO conjugates and were 6%, 36%, and 65% for BChE‐PCBAM‐IO conjugates. BChE‐polymer‐oxime conjugates were purified by ultrafiltration (100 kDa cut‐off membrane). Not surprisingly, higher ratios of alkyne‐imidazolium‐oxime resulted in higher stoichiometries of oximes per BChE monomer (15, 44, and 69 for BChE‐PDMAA‐IO conjugates and 8, 49, and 90 for BChE‐PCBAM‐IO conjugates). The significant increases in molecular weights for the BChE‐PDMAA‐IO and BChE‐PCBAM‐IO conjugates were also observed with UV–vis spectrum and SDS‐PAGE (Figure S4, Supporting Information). It should be noted that the polymer length was not varied in each conjugate, just the ratio of oxime to free azide within the same polymer chains.

### 2.2 BChE‐Polymer‐Azide and BChE‐Polymer‐Oxime Conjugate Activity

We next measured the functional activities of the BChE conjugates using Michaelis–Menten kinetics. The turnover number (*k*_cat_), Michaelis constant (*K*_M_), and overall catalytic efficiency (*k*_cat_/*K*_M_) for BChE, BChE‐Br, and BChE‐polymer conjugates were determined using acetylthiocholine iodide (ATC) as a substrate in 50 × 10^−3^ m sodium phosphate buffer (pH 7.4, room temperature) (**Table** **2**). BChE‐Br retained 93% activity compared to native BChE, demonstrating that initiator modification did not significantly impact function. Polymer growth of PDMAA and PCBAM decreased activity to approximately 67% for BChE‐PDMAA‐N_3_ and 54% for BChE‐PCBAM‐N_3_. Further reductions in activity were observed after clicking the oxime to generate the BChE‐PDMAA‐IO and BChE‐PCBAM‐IO conjugates. BChE‐polymer‐oxime conjugates retained 22%, 9%, and 3% of activity for BChE‐PDMAA‐IO_15_, BChE‐PDMAA‐IO_44_, and BChE‐PDMAA‐IO_69,_ respectively, and 26%, 22%, and 17% of activity for BChE‐PCBAM‐IO_8_, BChE‐PCBAM‐IO_49_, and BChE‐PCBAM‐IO_90_, respectively. The activity of native BChE in the presence of the same concentration of free alkyne‐imidazolium‐oxime (70‐fold) was similar to the native enzyme (3.5 times higher than the BChE‐PDMAA‐IO_69_ conjugate at 1 × 10^−9^ m BChE concentration) (Figure S5A, Supporting Information). Activity loss was essentially proportional to the degree of oxime content, but was more noticeable in the BChE‐PDMAA‐IO conjugates. The activity loss was due to slight decreases in *k*_cat_ and significant increases in *K*_M_ (*K*_M_ of BChE‐PDMAA‐N_3_ was 50 ± 13 × 10^−6^ m and increased to 82 ± 20, 130 ± 53, and 338 ± 100 × 10^−6^ m with 15, 44, 69 oximes, respectively). For BChE‐PCBAM‐IO, there were slight decreases in *k*_cat_, but no oxime content dependent change in *K*_M_. Although protein–polymer conjugates often experience activity losses, we hypothesized that an oxime‐induced effect could explain the observed data. Oximes function as reactivators by interacting directly with inhibitors in the active site. We therefore measured whether the conjugated oximes were acting as competitive inhibitors, which would decrease apparent conjugate activity as the number of oximes per polymer chain increased. We observed increased *K*_M_ as oxime content increased in the BChE‐PDMAA‐IO conjugates. The hydroxyl groups of oximes can only reactivate BChE active sites in their deprotonated form (negatively charged oximate).^76^ Additionally, the positively charged nitrogen in the imidazolium ring is known to significantly affect the affinity of tertiary and quaternary oximes to the anionic region at the BChE active site.^24^ The p*K*_a_ of the alkyne‐imidazolium‐oxime, determined spectroscopically, was 8.10 (Figure S6, Supporting Information)^25^ (similar to that of a related imidazolium oxime analog 37 (p*K*_a_ 8.27)).^24^ If competitive inhibition by oximes was occurring, then one would not expect to see the effect below the oxime's p*K*_a_. We determined reaction rates at a single substrate concentration for BChE‐polymer conjugates at pH 6.0 and 7.4 and compared them to the activity of native BChE (Figure S5, Supporting Information). Oxime‐free BChE‐PDMAA‐N_3_ retained 84% activity at pH 7.4 and 91% activity at pH 6.0. However, the activity retention of BChE‐PDMAA‐IO_69_ increased from 26% at pH 7.4 to 77% at pH 6.0. These data could be explained by polymer‐conjugated oximes competitively inhibiting the enzyme above their p*K*_a_. Interestingly, Primozic and co‐workers have estimated the affinity of a large number of imidazolium‐oximes to BChE^24^ and have shown that imidazolium‐oximes can bind to the BChE active site more efficiently than classical pyridinium‐oximes.^25^ PDMAA‐IO had an IC_50_ of 1 × 10^−6^ m (Figure S7, Supporting Information), which was similar to the imidazolium‐oximes reported by Primozic and co‐workers.^24^ Increasing the oxime content in BChE‐PCBAM‐IO conjugates did not affect substrate affinities (*K*_M_). The structures for the substrate (ATC) and monomer (CBAM) are similar in that they both contain a positively charged quaternary ammonium. ATC uses this positive charge to electrostatically interact with the anionic region of BChE's active site. This led us to consider whether CBAM side chains, in addition to the positively charged oximes, might competitively inhibit BChE. Since the CBAM to oxime ratio in the polymer chains was high (≈5% oximes), increased oxime content did not change the apparent *K*_M_. Overall, our data showed that BChE‐polymer‐oxime conjugates were active and that the oxime side chains interacted with the enzyme active site, possibly acting as competitive inhibitors.

**Table 2:** Michaelis–Menten kinetic parameters

	*K*_M_ [× 10^−6^ m]	*V*_max_ [× 10^−6^ m min^−1^]	*k*_cat_ [min^−1^]	*k*_cat_/*K*_M_ [min^−1^ (× 10^−6^ m) ^−1^]
BChE	43 ± 12	9.8 ± 0.7	19.6 ± 1.4 × 10^3^	455 ± 68
BChE‐Br	38 ± 10	8.1± 0.5	16.2 ± 1.0 × 10^3^	426 ± 56
BChE‐PDMAA‐N_3_	50 ± 13	7.3 ± 0.5	15.6 ± 1.0 × 10^3^	312 ± 41
BChE‐PCBAM‐N_3_	46 ± 8	5.2 ± 0.2	10.4 ± 4.0 × 10^3^	226 ± 16
BChE‐PDMAA‐IO_15_	82 ± 20	4.0 ± 0.3	8.0 ± 0.6 × 10^3^	98 ± 13
BChE‐PDMAA‐IO_44_	130 ± 53	2.5 ± 0.3	5.0 ± 0.6 × 10^3^	38 ± 11
BChE‐PDMAA‐IO_69_	338 ± 100	2.3 ± 0.3	4.6 ± 0.6 × 10^3^	14 ± 3
BChE‐PCBAM‐IO_8_	95 ± 21	5.3 ± 0.3	10.6 ± 0.6 × 10^3^	112 ± 12
BChE‐PCBAM‐IO_49_	90 ± 30	4.1 ± 0.4	8.2 ± 0.8 × 10^3^	91 ± 19
BChE‐PCBAM‐IO_90_	99 ± 28	3.6 ± 0.5	7.2 ± 1.0 × 10^3^	72 ± 16

Acetylthiocholine (ATC) was used as a BChE substrate at the range of 1–700 × 10^−6^ m, BChE concentration was 0.5 × 10^−9^ m, DTNB concentration was 0.1 × 10^−3^ m, and 50 × 10^−3^ m phosphate buffer pH 7.4 was used at room temperature. Parameters were calculated using the Michaelis–Menten model fitting feature in GraphPad Prism 5 for Windows (GraphPad Software, San Diego, CA).

### 2.3 Inhibition of BChE‐Polymer‐Oxime Conjugates by Paraoxon

**Figure 3:**
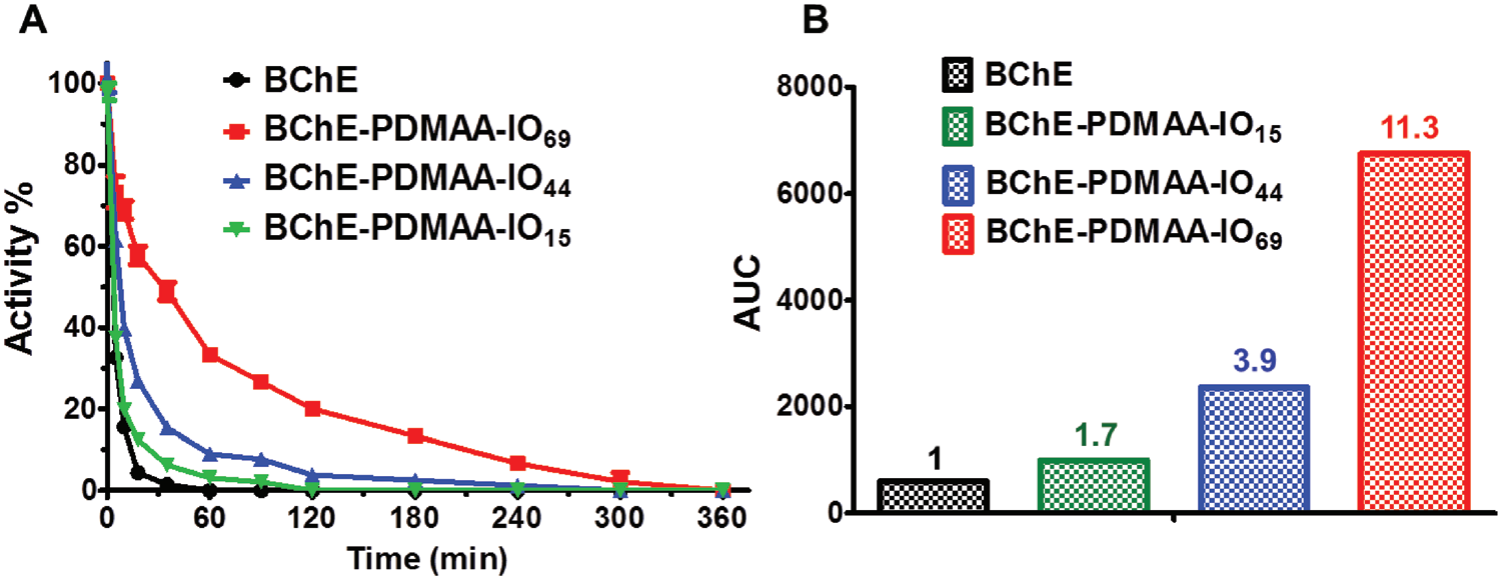
A) Inhibition assay of BChE and BChE‐PDMAA‐IO conjugates by 3.2‐fold stoichiometric excess of POX at pH 7.4 for 360 min. Results are presented as mean values ± standard deviation (*n* = 3). B) Area under the curves in subplot (A).

Our original interest was in whether oxime containing polymers could reactivate OP‐inhibited BChE. Since we had already observed that oxime polymers could interact with the active site of BChE, we next determined whether the oxime containing polymers might also simply protect BChE from inactivation by OP. Using paraoxon (POX) as a toxic organophosphate, we exposed BChE (20 × 10^−9^ m) to a 3.2‐fold stoichiometric excess of POX at pH 7.4 and measured activity loss over time. Unsurprisingly, oxime‐free BChE‐PDMAA‐N_3_ and BChE‐PCBAM‐N_3_ conjugates did not protect BChE against POX‐induced inactivation (Figure S8, Supporting Information). BChE‐PCBAM‐IO conjugates were minimally protected against inhibition by POX (Figure S9, Supporting Information), but the BChE‐PDMAA‐IO conjugates required considerably more POX to inactivate the enzyme (**Figure** **3**). The ability of BChE‐PDMAA‐IO to resist inactivation by POX increased with the amount of oxime in the polymer chains. Following inhibition by POX, BChE‐PDMAA‐IO_15_, BChE‐PDMAA‐IO_44_, and BChE‐PDMAA‐IO_69_ conjugates retained 6.2%, 13.2%, and 33.3% activity, respectively, (19, 41, and 103 times that of native BChE) (Figure 3) after 1 h. The BChE‐PDMAA‐IO_69_ conjugate lost 100% activity after 6 h. It is known that native BChE scavenges POX in a one‐to‐one ratio which means that the number of turnovers possible of unmodified BChE for an inhibitor would be 1. If we assume that the rate of reactivation was the same for native BChE and the BChE‐PDMAA‐IO conjugates, then the maximum potential number of possible turnovers that a single enzyme molecule could generate for BChE‐PDMAA‐IO_15_, BChE‐PDMAA‐IO_44_, and BChE‐PDMAA‐IO_69_ conjugates would be 1.7, 3.9, and 11.3, respectively, based on the area under the curves in Figure 3. Nevertheless, the dependence of degree of protection on oxime‐enzyme stoichiometry could be explained by a combination of three mechanisms. First, the oxime side chains could have been competing with POX for binding to the active site, which would slow the rate of inhibition as oxime concentration was increased. Thus, the reversible interaction of the positively charged imidazolium rings with the anionic region of BChE's active site would restrict the accessibility of POX to the active site serine. Second, the oxime side chains could have reacted directly with POX before it had the chance to reach the active site. Finally, the conjugated oxime polymers could have reactivated the inhibited BChE‐conjugate either inter‐ or intramolecularly as described above. Intramolecular reactivation would occur when oxime containing polymers reactivated the same molecule of BChE that they were covalently attached to. Intermolecular reactivation would occur when oxime containing polymers covalently attached to one BChE would reactivate the active site of a neighboring BChE‐conjugate. We therefore designed a series of experiments to elucidate the degree to which each of these mechanisms might have been contributing to the observed protective effects within BChE‐PDMAA‐IO conjugates.

We first explored whether or not oxime side chains were directly reacting with soluble POX. The reaction between POX and oximes was monitored spectroscopically (Figure S10, Supporting Information). POX (20 × 10^−6^ m) was mixed with NH_2_‐PEG_3_‐IO (140 × 10^−6^ m) at either pH 6.0 or 8.0, and no reaction was detected after 24 h at room temperature. These data ruled out that the increase in the time for POX to fully inhibit the conjugate was not caused by a direct reaction between oxime and POX.

### 2.4 Reactivation of Paraoxon‐Inhibited BChE‐Polymer‐Oxime Conjugates

Next, we designed experiments that would determine whether the polymer‐oximes could elicit intramolecular and/or intermolecular reactivation of a covalent BChE‐nerve agent complex. Moreover, if reactivation was observed, we wanted to determine which pathway (intra‐ or intermolecular) was dominant. We first determined whether free alkyne‐imidazolium‐oxime was an effective reactivator of BChE‐POX complexes. Oxime‐induced reactivation begins with the approach of the oxime to the inhibited BChE active site, followed by nucleophilic attack of the oxime on the diethyl‐phosphoryl moiety in POX‐inhibited BChE. More than 20% of the activity of POX‐inhibited BChE was recovered after the addition of a large stoichiometric 25 000‐fold excess of free alkyne‐imidazolium‐oxime (0.5 × 10^−3^ m) at pH 8.0 over 2 h (Figure S11, Supporting Information). The enzyme was also reactivated by a positive control reagent, 2‐PAM (25000‐fold, 0.5 × 10^−3^ m), with restoration of 44% activity over the same time period.

We were curious as to whether the polymeric versions of alkyne‐imidazolium‐oxime would have the same reactivating abilities as their precursors, so we selected the BChE‐PDMAA‐IO_69_ conjugate for further studies since it had the highest degree of apparent protection against POX inactivation. At pH 6.0, two pH units below the p*K*_a_, the oximate form of oximes are only 1% of the protonated oxime concentration. We predicted, therefore, that at low pH we would be able to inactivate the conjugates with POX without reactivation interference by the polymer‐oxime side chains. We determined that the rate of inhibition of BChE‐PDMAA‐IO_69_ by POX was almost the same as with native BChE at pH 6.0 confirming that oximate‐induced protection of the enzyme against POX had been prevented at this lower pH (Figure S12, Supporting Information). OP‐induced complete inactivation was achieved by mixing a tenfold molar excess of POX with BChE‐PDMAA‐IO_69_ or free BChE at pH 6.0 for 10 min. The POX‐BChE was then purified by removing excess POX with ultrafiltration, followed by a 50‐fold dilution in sodium phosphate buffer at pH 8.0 (or in negative control experiments, pH 6.0).

**Figure 4:**
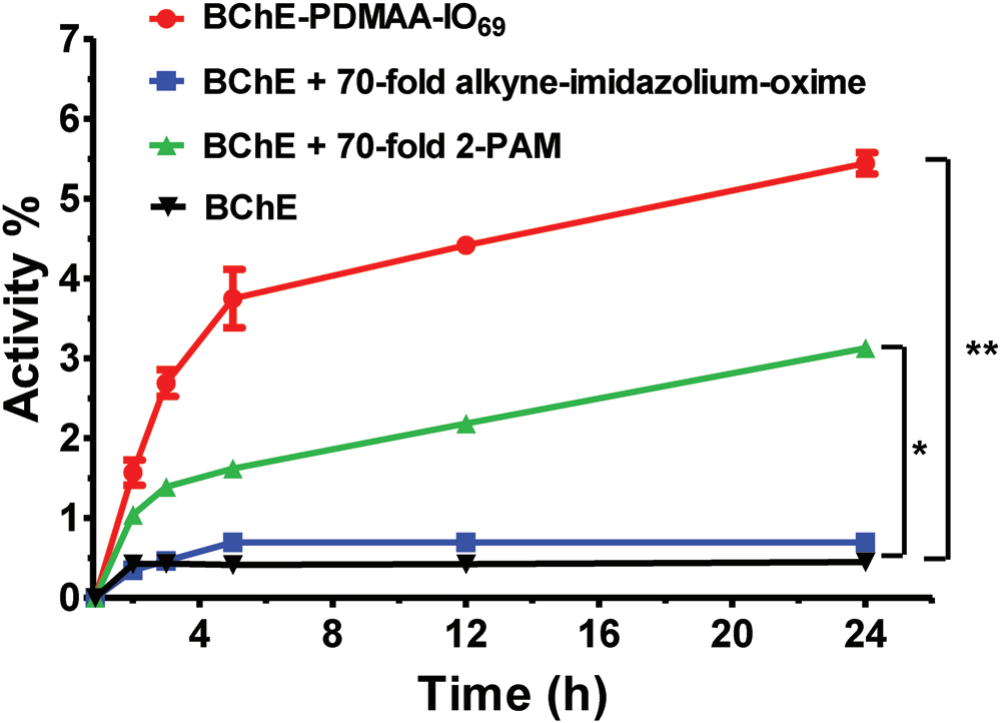
Reactivation assay of BChE and BChE‐PDMAA‐IO_69_ conjugate. 1 × 10^−6^ m BChE‐PDMAA‐IO_69_ or BChE were inhibited by tenfold excess of POX at pH 6.0 and then diluted 50‐fold at pH 8.0. Statistics (Student's *t*‐test) was performed by comparing each treatment group with the corresponding native BChE group (*p < 0.05, **p < 0.01). Results are presented as mean values ± standard deviation (*n* = 3).

Reactivation of POX‐BChE was subsequently measured by incubating POX‐BChE at either pH 6.0 or 8.0. After mixing the assay components, we removed aliquots at specified time intervals and measured remaining activity of the enzyme by addition of ATC at either pH 6.0 or 8.0. Control experiments were performed with native POX‐BChE in the presence of oximes (2‐PAM or alkyne‐imidazolium‐oxime) at the same stoichiometry as in the BChE‐polymer‐oxime conjugate. Degree of reactivation was calculated relative to the respective initial activities of either free BChE or the conjugate. After 24 h at pH 8.0, the BChE‐PDMAA‐IO_69_ conjugate recovered 6% of activity, which was more than ten times the activity of inhibited native BChE in the presence of 70 equivalents of free alkyne‐imidazolium‐oxime (**Figure** **4**). Our initial studies had shown that the alkyne‐imidazolium‐oxime was not as effective as a reactivator as 2‐PAM, but the BChE‐PDMAA‐IO_69_ was twice as effective as native BChE in the presence of a 70‐fold molar excess of 2‐PAM (Figure 4). There was no significant difference of activity recovery between BChE‐PDMAA‐IO_69_ conjugate and BChE control groups at pH 6.0 (Figure S13, Supporting Information).

We next studied how much free alkyne‐imidazolium‐oxime would be needed to reactivate the same amount of native POX‐BChE. We found that a more than 325‐fold molar excess of the free alkyne‐imidazolium‐oxime had the same reactivation potency as BChE‐PDMAA‐IO_69_ (Figure S14, Supporting Information). Accumulating the oximes around the surface of the enzyme had an activation effect that was equivalent to increasing the apparent concentration of the oximate by at least fivefold. We also showed that the conjugate could be reactivated successfully by a large excess of 2‐PAM (25000‐fold) at pH 6.0 and 8.0 (Figure S15, Supporting Information). Simple reactivation would not be the only possible explanation of why we observed an increase in activity over time after complete inactivation of the BChE‐oxime conjugates. Some of the observed reactivation of BChE‐PDMAA‐IO_69_ may have been due to subtle conformational changes of the BChE active site induced by the conjugated oxime‐polymer. This could have caused the release of small amounts of POX without a direct reaction of the oxime with the phosphorous atom of POX. In addition, it was possible that small amounts of residual POX in the conjugates that were prepared at pH 6 were able to keep inhibiting the native enzyme, but that the conjugates were inactivated less rapidly. Although we were unable to rule out either of these possible side reactions, both seem unlikely to be prominent causes of the observed reactivation.

**Figure 5:**
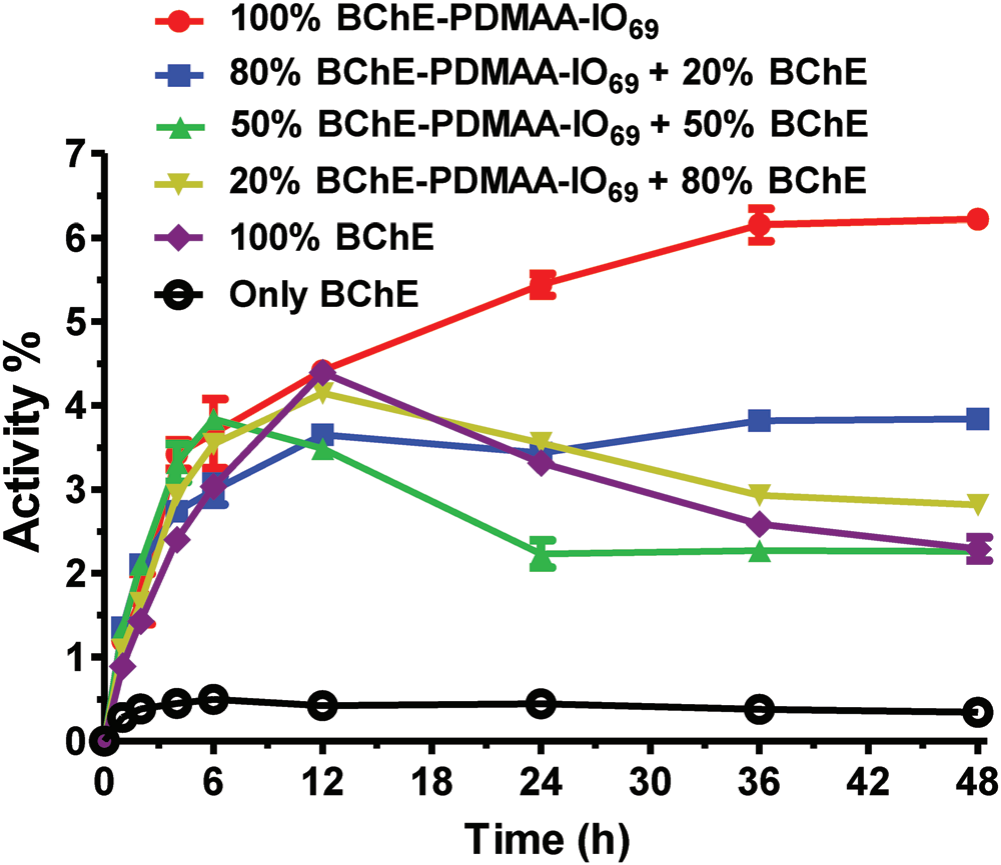
Reactivation assay of the mixtures of BChE, BChE‐PDMAA‐IO_69_, and PDMAA‐IO (Oxime/BChE = 70). 1 × 10^−6^ m protein of the mixture of BChE, BChE‐PDMAA‐IO_69_ was inhibited by tenfold excess of POX at pH 6.0 and then diluted 50‐fold at pH 8.0. Results are presented as mean values ± standard deviation (*n* = 3).

To elucidate whether the apparent reactivation of BChE‐PDMAA‐IO_69_ was consistent with intramolecular and/or intermolecular interactions, mixtures of native BChE, BChE‐PDMAA‐IO_69_ and PDMAA‐IO were added in different ratios and reactivation was monitored over 48 h (**Figure** **5**). If only intramolecular interactions were inducing reactivation, then one would expect that replacing conjugated oximes with free polymeric oximes would eliminate the observed reactivation. Further, as the fraction of native enzyme increased, it would be unlikely to be reactivated by the conjugate. In the case of intermolecular reactivation, replacing the conjugated oximes with free polymeric oximes would result in some degree of reactivation of both native and conjugate in the mixture, but one would expect less oxime available at the active site leading to reduced inactivation. We kept the total oxime content constant at 70 equivalents, while we varied the fraction of oxime supplied by the conjugate and by free polymer‐oxime. BChE‐PDMAA‐IO_69_ with no free polymer‐oxime had the highest degree of observed reactivation (we use the term observed because, as explained above we could not determine the impact of side reactions). The degree of observed reactivation decreased steadily as the fraction of free polymer‐oxime increased. These data showed that there was substantial apparent intramolecular reactivation of BChE‐PDMAA‐IO‐POX conjugates. When we studied native BChE‐POX reactivation, we observed about 35% of the reactivation observed in BChE‐PDMAA‐IO‐POX. We surmised that although there was evidence of intermolecular reactivation, the majority of the observed effect was likely due to intramolecular reactivation.

## 3 Conclusion

We have described the synthesis of protein‐ATRP synthesized BChE‐polymer conjugates in which imidazolium‐oxime molecules were “clicked” to the polymers to generate functional BChE‐polymer‐oxime conjugates. The activity of this unique class of conjugates, at neutral or high pH, was dependent on the degree of oxime content in the polymer chains. The BChE‐polymer‐oxime conjugates exhibited an unusual delayed response to organophosphate‐induced inactivation, thereby extending the time that BChE could remain active in the presence of an OP inhibitor. After inhibiting the conjugates at a pH at which the polymer‐oximes were inactive and nonprotective, we were able to study auto‐reactivation. Evidence emerged that the majority of the BChE‐polymer‐oxime conjugate reactivation was intramolecular. We are now in a position to explore whether the attachment of next generation BChE‐OP reactivators to protein‐ATRP derived BChE‐polymer‐N_3_ conjugates might generate high performance self‐reactivating cholinesterases for decontamination and therapeutic use. Self‐reactivating cholinesterase conjugates would use the very weakness in biology that organophosphate nerve agents target, to bind and then destroy the agent in a victory for the polymer‐enhanced biomacromolecule.

## 4 Experimental Section

*Solvents and Reagents*: All solvents were purchased from Fisher Scientific (Pittsburgh, PA) and used as received. BChE was provided by Dr. Oksana Lockridge at the Eppley Institute, University of Nebraska Medical Center. The protein‐reactive ATRP initiator (NHS‐Br initiator) was synthesized the same way as previously described.^72^ Copper(II) sulfate (CuSO_4_), copper(II) bromide (CuBr_2_), triethylamine, sodium ascorbate (NaAsc), hydroxylamine hydrochloride, 2‐imidazolecarboxaldehyde, iodomethane, methacryloyl chloride, *N*‐(3‐(dimethylamino)propyl)acrylamide, 2,2‐diphenyl‐1‐picrylhydrazyl, 1,1,4,7,10,10‐ hexamethyltriethylenetetramine (HMTETA), oxetan‐2‐ylideneoxonium, propargyl bromide, ATC, S‐butyrylthiocholine iodide (BTC), 5,5′‐dithiobis (2‐nitrobenzoic acid) (DTNB), *O*,*O*‐diethyl *O*‐(4‐nitrophenyl) phosphate (Paraoxon, POX), and *N*,*N*‐dimethylacrylamide (DMAA) were purchased from Sigma‐Aldrich. DMAA and HMTETA were purified prior to use with a basic alumina column. Azide‐PEG‐Amine and 2‐(4‐((bis((1‐(tert‐butyl)‐1*H*‐1,2,3‐triazol‐4‐yl)methyl)amino)methyl)‐1*H*‐1,2,3‐triazol‐1‐yl) acetic acid (BTTAA) were purchased from Click Chemistry Tools LLC.

*Nuclear Magnetic Resonance (NMR) Analysis*: A 300 MHZ spectrometer (Bruker Avance), located in the Center for Molecular Analysis, Carnegie Mellon University, Pittsburgh, PA, was used for ^1^H NMR spectra analysis.

*UV–vis Spectrophotometry*: A UV–vis spectrophotometer (Lambda 45, PerkinElmer) was used to record UV–vis spectra. A plate reader (Bio‐Tek Synergy H1) was used for high‐throughput absorbance measurements.

*GPC*: The *M*_w_ and *Ð* were determined by GPC on a Waters 2695 Series system (Waters Ultrahydrogel Linear, 500 and 250) with 3‐columns, using sodium phosphate buffer (100 × 10^−3^ m, 0.01% sodium azide) as the eluent with a refractive index (RI) detector. The flow rate was 1.0 mL min^−1^. The samples were prefiltered by a 0.22 µm pore size filter. GPC calibration was based on poly(ethylene glycol) standards.

*Dynamic Light Scattering (DLS)*: DLS was performed on a Micromeritics (Norcross, GA) NanoPlus 3 dynamic light scattering instrument. The samples (1.0 mg mL^−1^, protein) were filtered by a 0.22 µm pore size filter before analysis. The instrument was used to measure the number (reported in supplemental information), volume (reported in supplemental information), and intensity (reported in the text) average hydrodynamic diameters (*D*_h_) of BChE, BChE‐Br, BChE‐PCBAM‐N_3_, and BChE‐PDMAA‐N_3_ conjugates.

*Electrospray Ionization Mass Spectrometry (ESI‐MS) Analysis*: ESI‐MS was measured by a quadrupole field ion trap mass spectrometer (Finnigan LCQ, Thermo‐Fisher). The samples were prepared at 100 × 10^−6^ m in MeOH.

*Protein Analysis by the Bicinchoninic Acid (BCA) Assay*: BChE conjugates (1.1 mg mL^−1^, BChE) were dissolved in sodium phosphate buffer (50 × 10^−3^ m, pH 7.4) and the samples (20 or 10 µL) were mixed with BCA working reagent solution (Reagent A/Reagent B = 50/1). The mixture was incubated for 15 min at 60 °C. Absorbance of each sample was recorded at 562 nm by a plate reader (Bio‐Tek Synergy H1). Protein concentrations of the conjugates were determined by comparing the absorbance to a standard curve (native BChE).

*Determination of BChE Active Site Concentration*: BChE (100 × 10^−9^ m) were incubated with 0.2, 0.3, 0.5, and 0.7‐fold of POX at room temperature. At 60 min, an aliquot was diluted 100 times in sodium phosphate buffer (50 × 10^−3^ m, pH 7.4) containing DTNB. Upon addition of ATC, residual enzyme activity was measured. The final concentrations of ATC and DTNB in the assay were 1.0 and 0.1 × 10^−3^ m, respectively. The activity of uninhibited BChE was used as 100% activity. All enzymatic activity measurements were performed in sodium phosphate buffer (50 × 10^−3^ m, pH 7.4) using the Ellman spectrophotometric method at 412 nm on a Lambda 2 PerkinElmer UV–vis spectrophotometer.^77^

*SDS‐PAGE Analysis*: A 10 µL aliquot of BChE, BChE‐Br, or BChE‐polymer solution (1.0 mg mL^−1^ protein in PBS) was mixed with 10 µL of 2× SDS‐PAGE loading buffer and heated at 95 °C for 10 min. The samples were loaded onto 4%–15% precast gel which was run at 200 V for 30 min. After washing with distilled water, the gel was stained with 50 mL PageBlue staining solution for 1 h and destained overnight by distilled water.

*Synthesis of 2‐((Hydroxyimino)methyl)‐3‐methyl‐1‐(prop‐2‐yn‐1‐yl)‐1H‐imidazol‐3‐ium Iodide*: 2‐((hydroxyimino)methyl)‐3‐methyl‐1‐(prop‐2‐yn‐1‐yl)‐1*H*‐imidazol‐3‐ium iodide (which is referred throughout as alkyne‐imidazolium‐oxime) was synthesized as described in the literature.^23^ In brief, to a mixture of 2‐imidazolecarboxaldehyde (11 mmol) and K_2_CO_3_ (22 mmol,) in 30 mL DMF, 3‐bromoprop‐1‐yne (22 mmol) was added and the reaction mixture was stirred at room temperature for 20 h. The reaction mixture was then filtered and the filtrate was diluted with 300 mL distilled water, and the resulting solution was extracted by ethyl acetate (150 mL × 4). The organic phase was dried by MgSO_4_ and removed by rotary evaporator to give alkylimidazole‐2‐carbaldehyde, 86% yield.

Hydroxylamine hydrochloride (13.5 mmol) was mixed with Na_2_CO_3_ (13.5 mmol) in 30 mL water and added by alkylimidazole‐2‐carbaldehyde (13.5 mmol). The reaction mixture was stirred at room temperature for 2.5 h. The product of alkylimidazole‐2‐carbaldehyde oxime was precipitated and collected by filtration, washing with distilled water, and dried over vacuum, 56% yield.

Iodomethane (33 mmol) in 40 mL MeOH and 3 mL DMSO was added with the alkylimidazole‐2‐carbaldehyde oxime (3.35 mmol). The reaction mixture was stirred at room temperature for 5 d. The alkyne‐imidazolium‐oxime product was purified by silica gel column (from EA to EA/MeOH = 5/1).Yellow powder, 40% yield. ^1^H NMR (300 MHz, MeOD‐d4) δ 8.45 (s, 1H, Ar H), 7.70 (d, *J* = 3, 1H, Ar H), 7.51 (d, *J* = 3, 1H, Ar H), 5.18 (d, *J* = 3, 2H; CH_2_), 3.91 (s, 3H; CH_3_), 2.99 (t, *J* = 3, 1H; CH). See Figure S16 in the Supporting Information.

*Synthesis of N‐(20‐azido‐3,6,9,12,15,18‐hexaoxaicosyl)methacrylamide Monomer (MA‐PEG_6_‐N_3_)*: N_3_‐PEG_6_‐NH_2_ (1.43 mmol) and Et_3_N (2 mmol) were added to 10 mL of DCM and cooled in a flask by an ice‐bath. Methacryloyl chloride (2 mmol) in 10 mL DCM was added drop‐wise into the mixture. The reaction was slowly warmed to room temperature under stirring overnight. The reaction mixture was washed with 1 m HCl (25 mL × 1), NaHCO_3_ (sat. 25 mL × 1), and brine (sat. 25 mL × 1), the organic phase was then dried with MgSO_4_. The solvent was removed by rotary evaporator. The product was purified by silica gel (EA/Hex: from 1/1 to 5/1). Colorless oil, 60% yield. ^1^H NMR (300 MHz, CDCl_3_) δ 6.40 (s, 1H; NH), 5.72 (s, 1H; CH), 5.35−5.33 (m, 1H; CH), 3.71−3.60 (m, 24H; 12CH2), 3.56−3.51 (m, 2 H; CH2), 3.42−3.39 (m, 2 H; CH2), 1.98 (s, 3H; CH3). MS (ESI) *m*/*z*: [M + Na]^+^ calcd for C_18_H_34_N_4_O_7_Na^+^ 441.2; found m/z 441.3. See Figure S16 in the Supporting Information.

*Synthesis of 3‐((3‐Acrylamidopropyl)dimethylammonio)propanoate (Carboxybetaine Acrylamide, CBAM): N*‐(3‐(dimethylamino)propyl)acrylamide (20 mmol) and 2,2‐diphenyl‐1‐picrylhydrazyl (8 µmol) were dissolved in 10 mL tetrahydrofuran (THF) and cooled to 0 °C in an ice‐bath. Oxetan‐2‐one (25 mmol) in 20 mL THF was added drop‐wise into the mixture over 1.5 h in the ice‐bath. The reaction mixture was stirred at 4 °C for 36 h. The white precipitate was filtered‐off and washed with THF and ether. The precipitate was dissolved in water and freeze‐dried to get a white powder, 75% yield. ^1^H NMR (300 MHz, D_2_O) δ 6.20−6.16 (m, 2H; CH2), 5.72 (dd, *J* ^1^ = 0.9, *J* ^2^ = 0.3, 1H; CH), 3.51 (t, *J* = 0.9, 2H; CH2), 3.35−3.26 (m, 4 H; 2CH2), 3.02 (s, 6H; 2CH3), 2.60 (t, *J* = 0.9, 2H; CH2), 2.05−1.95 (m, 2 H; CH2). See Figure S16 in the Supporting Information.

*Synthesis of NH_2_‐PEG_3_‐IO*: Sodium ascorbate (NaAsc, 0.1 mmol) was added into CuSO_4_ solution (100 × 10^−3^ m × 300 µL, 0.03 mmol) with BTTAA (0.03 mmol, in 300 µL H_2_O). The mixture was added to alkyne‐imidazolium‐oxime (see Figure S17 in the Supporting Information) (0.1 mmol) and N_3_‐PEG_3_‐NH_2_ (0.1 mmol) in 600 µL DMF. The reaction mixture was incubated at room temperature overnight. The product was purified by silica gel (from EA to EA/MeOH = 5/1) and dried by vacuum. Dark oil, 76% yield. ^1^H NMR (300 MHz, CDCl_3_) δ 8.35 (s, 1H; CH), 8.09 (s, 1H, Ar H), 7.52 (s, 1H, Ar H), 7.45 (s, 1H, Ar H), 5.64 (s, 2H; CH2), 3.95−3.89 (m, 2H; CH2), 3.86 (s, 3H; CH3), 3.70−3.67 (m, 2H; CH2), 3.58−3.52 (m, 10H; 5CH2), 3.16−3.12 (m, 2 H; CH2). NH_2_‐PEG_3_‐IO was used as a standard for the calculation of oxime/BChE ratios.

*Synthesis of BChE‐Polymer‐Oxime Conjugates*: Synthesis of the ATRP initiating molecules (BChE‐Br) was carried as described previously.^72^ Briefly, BChE (0.26 µmol) in 10 mL PBS buffer was buffer exchanged with sodium phosphate buffer (50 × 10^−3^ m, pH 8.0) by ultrafiltration (100 kDa cut‐off membrane) three times to total volume of 5 mL. Initiator (NHS‐Br, 19 µmol) in 200 µL DMSO was added to BChE solution and shaken at room temperature for 2 h. The excess NHS‐Br was removed by ultrafiltration (100 kDa cut‐off membrane) three times with sodium phosphate buffer (50 × 10^−3^ m, pH 7.4). The ratio of initiator to BChE was determined with a ninhydrin assay.^74^

To synthesize BChE‐PDMAA‐N_3_ conjugates, DMAA (108 µmol), MA‐PEG_6_‐N_3_ (4.3 µmol) and BChE‐Br (15 nmol) were mixed in 2 mL of sodium phosphate buffer (50 × 10^−3^ m, pH 7.4), sealed, and bubbled with argon at room temperature for 30 min. Next, deoxygenated catalyst‐ligand solution containing HMTETA (3.6 µmol), CuBr_2_ (3 µmol), and NaAsc (0.3 µmol) in distilled water (1 mL) were added to the initiator‐monomer solution, sealed, and stirred at room temperature for 2 h. BChE‐PDMAA‐N_3_ conjugate was purified by ultrafiltration (100 kDa cut‐off membrane) with sodium phosphate buffer (50 × 10^−3^ m, pH 7.4). BChE‐PCBAM‐N_3_ conjugate was synthesized by the same method, but the DMAA monomer was replaced by CBAM monomer. BChE, BChE‐Br, BChE‐PDMAA‐N_3_, and BChE‐PCBAM‐N_3_ conjugates were analyzed by GPC, NMR, and SDS‐PAGE.

Alkyne‐imidazolium‐oxime was attached to the polymer by alkyne‐azide cycloaddition “click” reaction.^75^ CuSO_4_ (10 × 10^−3^ m × 1.2 µL), NaAsc (10 × 10^−3^ m × 1.2 µL), and BTTAA (10 × 10^−3^ m × 2.4 µL) in distilled water were added into a BChE‐PDMAA‐N_3_ or BChE‐PCBAM‐N_3_ (28 × 10^−6^ m BChE, 430 µL) solution and then mixed with alkyne‐imidazolium‐oxime (Figure S17, Supporting Information) in distilled water at different ratios (oxime/BChE; 20, 60, or 120). The mixture was incubated at room temperature for 3 h. The BChE‐polymer‐oxime conjugates were purified by ultrafiltration (50 kDa cut‐off membrane) four times. The oximes to BChE ratios of the conjugates were determined by BCA assay and by BChE and NH_2_‐PEG_3_‐IO standard working curves (Figure S18, Supporting Information). The conversion of azide groups are 4%, 13%, and 20% for BChE‐PDMAA‐IO_15_, BChE‐PDMAA‐IO_44_, and BChE‐PDMAA‐IO_69_ conjugates, respectively and 6%, 36%, and 65% for BChE‐PCBAM‐IO_8_, BChE‐PCBAM‐IO_49_, and BChE‐PCBAM‐IO_90_ conjugates, respectively. BChE‐polymer‐oxime conjugates were analyzed by SDS‐PAGE and UV–vis spectrum.

*Polymer Cleavage from Conjugates*: PDMAA‐N_3_ from BChE‐PDMAA‐N_3_ were prepared by subjecting the corresponding conjugates (protein concentration 1.5 mg mL^−1^) to proteinase K (1 mg mL^−1^) in digestion buffer (10 × 10^−3^ m Tris·HCl, 2 × 10^−3^ m CaCl2, pH 7.4) at 60 °C for 24 h. The cleaved polymers were purified by ultrafiltration (50 kDa cut‐off membrane) three times. BChE−PCBAM‐N_3_ conjugates (2 mg protein) were placed in hydrolysis tubes and dissolved in 6 m HCl (2 mL). After five freeze−pump−thaw cycles, the hydrolysis was performed at 110 °C under a vacuum for 24 h. The cleaved polymers were purified by ultrafiltration (50 kDa cut‐off membrane) with distilled water, and were then lyophilized. The molecular weight and dispersity of the polymers were measured by GPC.

*Michaelis−Menten Kinetics*: ATC was used as a substrate to determine the functionality of BChE at room temperature. 1 mL of ATC (0.001 ≈ 0.7 × 10^−3^ m) and Ellman assay reagent DTNB (0.1 × 10^−3^ m) in 50 × 10^−3^ m sodium phosphate buffer (pH 7.4) were added to a 1.5 mL cuvette. Native BChE or BChE‐conjugate (5 × 10^−9^ m protein, 100 µL) was then added to the cuvette^77^ and the initial rate of substrate hydrolysis was measured by recording the increase in absorbance at 412 nm from TNB (extinction coefficient of 14 000 M^−1^ cm^−1^) using a Lambda 2 PerkinElmer UV–vis spectrophotometer. Michaelis−Menten parameters were determined using nonlinear curve fitting of initial hydrolysis rate versus substrate concentration in GraphPad 5.

*Inhibition Assay of BChE and BChE‐Conjugates*: BChE or BChE‐conjugates (20 × 10^−9^ m) were incubated with 3.2‐fold stoichiometric excess of POX at room temperature. At specified time intervals, an aliquot was diluted 20 times in sodium phosphate buffer (50 × 10^−3^ m, pH 7.4) containing DTNB. Upon addition of ATC, residual enzyme activity was measured. The final concentrations of ATC and DTNB in the assay were 1.0 and 0.1 × 10^−3^ m, respectively. The activity of uninhibited BChE or BChE‐conjugates was used to determine the time zero point. All enzymatic activity measurements were performed in sodium phosphate buffer (50 × 10^−3^ m, pH 7.4) using the Ellman spectrophotometric method at 412 nm on a Lambda 2 PerkinElmer UV–vis spectrophotometer.^77^

*Reactivation of BChE and BChE‐Conjugates*: BChE or BChE‐conjugates (1 × 10^−6^ m) were incubated with a tenfold molar excess of POX at pH 6.0 until complete inhibition was observed (10 min). The inhibited enzyme or conjugate was transferred into an ultrafiltration tube (50 kDa cut‐off membrane) to remove excess POX (10 000 × *g* for 5 min, three times). The enzyme or conjugate was diluted 50‐fold in sodium phosphate buffer (50 × 10^−3^ m, pH 6.0 or 8.0) with or without reactivators (2‐PAM, 500 × 10^−6^ m; alkyne‐imidazolium‐oxime, from 1.4 × 10^−6^ m to 500 × 10^−6^ m). At specified time intervals, the activities of the samples were measured by Ellman assay. The final concentrations of ATC and DTNB in the assays were 1.0 and 0.1 × 10^−3^ m, respectively. The activity of uninhibited BChE or BChE‐conjugate was used to determine the 100% activity.

*Reactivation of BChE, BChE‐PDMAA‐IO_69_*, *and PDMAA‐IO Mixtures*: A mixture of BChE, BChE‐PDMAA‐IO_69_, and PDMAA‐IO with different ratios (protein 1 × 10^−6^ m; Oxime/BChE = 70) were incubated with a tenfold excess of POX at pH 6.0 until complete inhibition was observed (10 min). Such inhibited mixtures were transferred into ultrafiltration tube (50 kDa cut‐off membrane) to remove excess POX (10 000 × *g* for 5 min, three times). The mixtures were diluted 50‐fold in sodium phosphate buffer (50 × 10^−3^ m, pH 8.0). At specified time intervals, the activities of the mixtures were measured by Ellman assay. Final concentrations of BTC and DTNB in the assay were 1.0 and 0.1 × 10^−3^ m, respectively. The activities of uninhibited mixtures were used to determine the 100% activity.

*pK_a_ of Alkyne‐Imidazolium‐Oxime*: The UV–vis spectrum of alkyne‐imidazolium‐oxime was measured with a Lambda 2 PerkinElmer UV–vis spectrophotometer (Figure S6A, Supporting Information). Protonation of oxime groups were monitored by UV–vis spectrophotometry using a Bio‐Tek Synergy H1 plate reader. The proton dissociation constants were determined from pH‐absorbance profiles at 270 nm.^25^ The p*K*_a_ of alkyne‐imidazolium‐oxime (Figure S6B, Supporting Information) was similar to that for the imidazolium‐oxime analog described in the literature.^24^

## Conflict of Interest

The authors declare no conflict of interest.

## Supporting information

Supporting Information
