# Supplementary material for: Tuning Butyrylcholinesterase Inactivation and Reactivation by Polymer‐Based Protein Engineering
Source: Adv Sci (Weinh). 2019 Nov 13;7(1):1901904. doi: 10.1002/advs.201901904 (PMC6947490; doi:10.1002/advs.201901904)
Supplement: Supplementary file 1 — Supporting Information [file ADVS-7-1901904-s001.pdf]

Copyright WILEY-VCH Verlag GmbH & Co. KGaA, 69469 Weinheim, Germany, 2019.

Supporting Information

for *Adv. Sci.,* DOI: 10.1002/advs.201901904

Tuning Butyrylcholinesterase Inactivation and Reactivation by Polymer-Based Protein Engineering

*Libin Zhang, Stefanie L. Baker, Hironobu Murata, Nicholas Harris, Weihang Ji, Gabriel Amitai, Krzysztof Matyjaszewski, and Alan J. Russell**

Supporting Information

Tuning Butyrylcholinesterase Inactivation and Reactivation by Polymer-Based Protein Engineering

*Libin Zhang, Stefanie L. Baker, Hironobu Murata, Weihang Ji, Gabriel Amitai, Krzysztof Matyjaszewski, and Alan J. Russell**

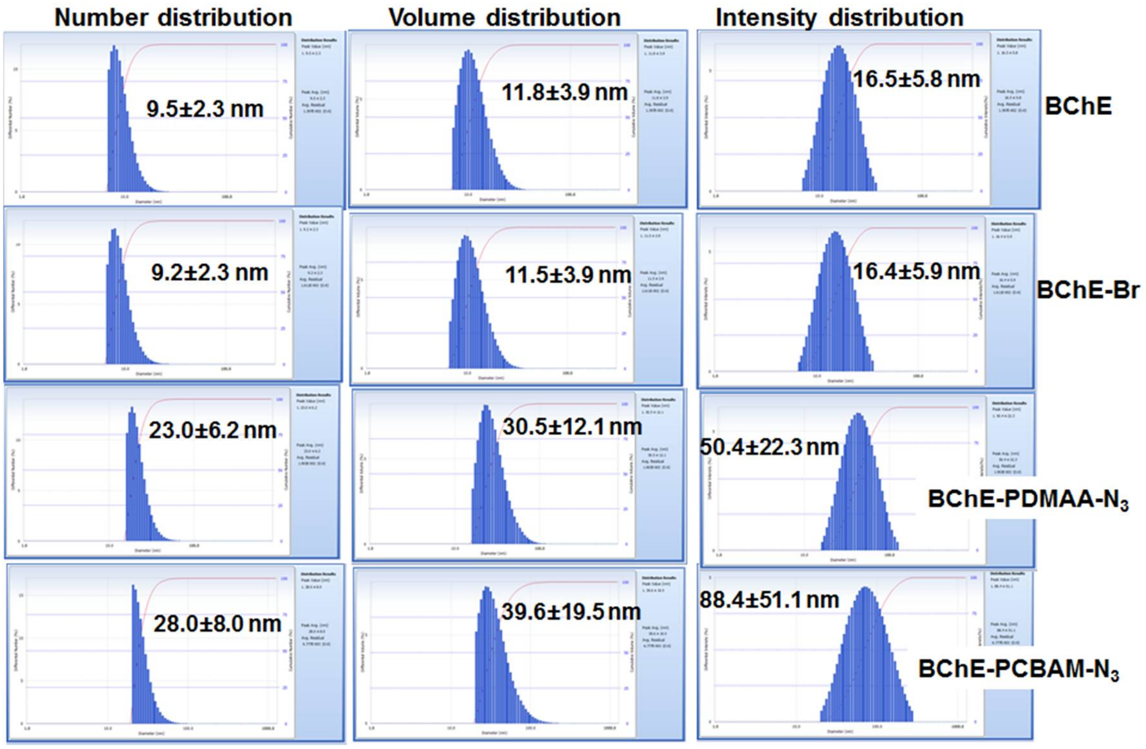

Figure S1. DLS analysis of BChE, BChE-Br, BChE-PDMAA-N_3 and BChE-PCBAM-N_3 conjugates

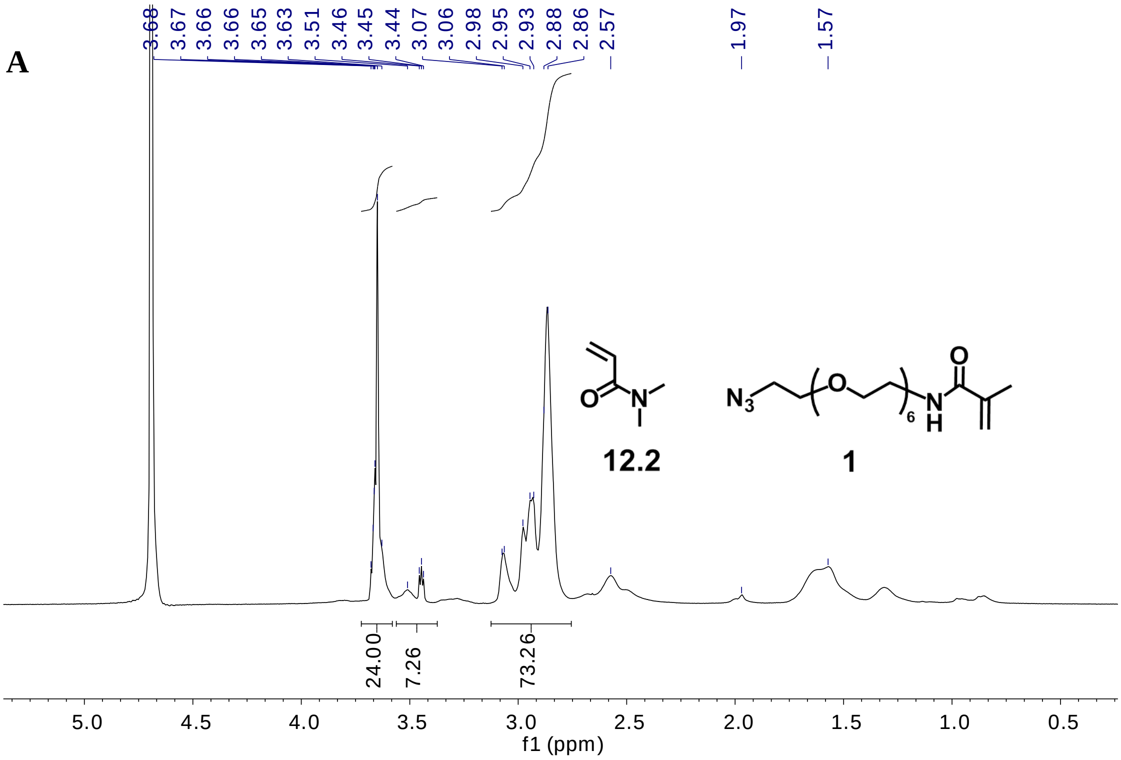

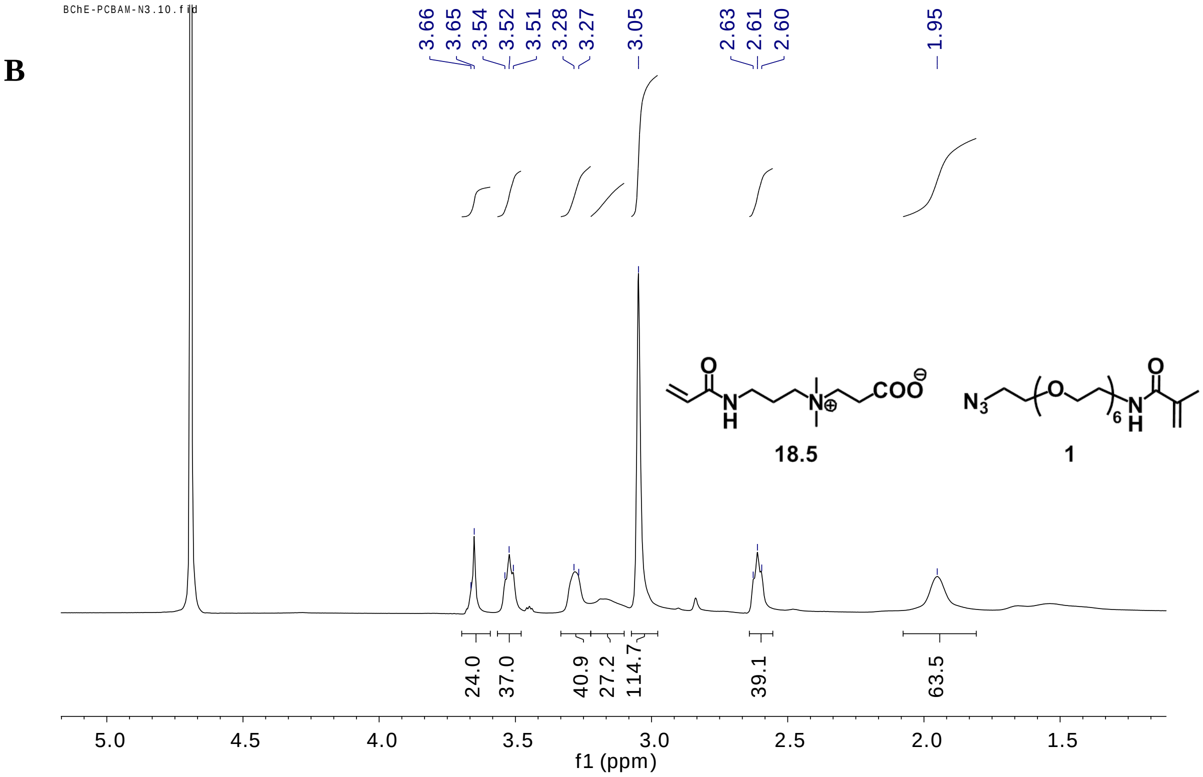

Figure S2. A) NMR analysis of BChE-PDMAA-N_3 conjugate, B) NMR analysis of BChE-PCBAM-N_3 conjugate

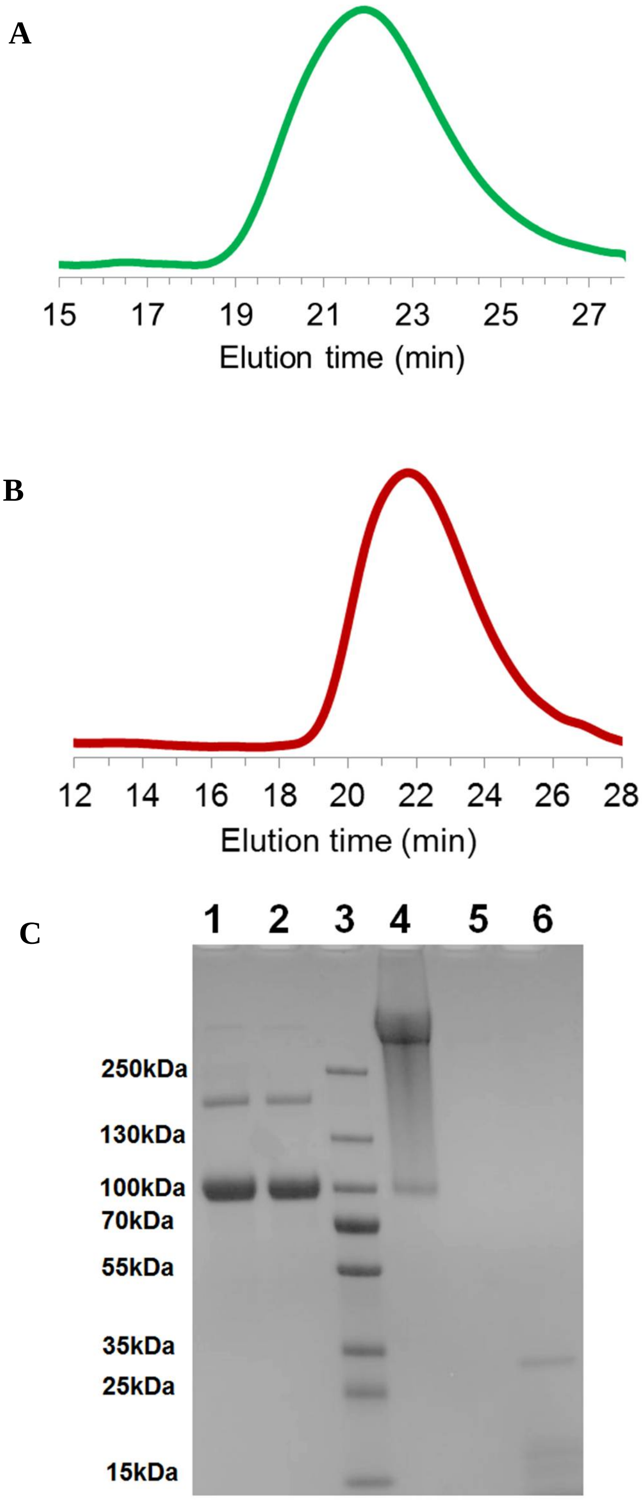

Figure S3. A) GPC analysis of PCBAM-N_3 from BChE-PCBAM-N_3 conjugate, B) GPC analysis of PDMAA-N_3 from BChE-PDMAA-N_3 conjugate, C) SDS-PAGE analysis of BChE-PDMAA-N_3 conjugate digestion by Proteinase K. Lane 1: BChE; Lane 2: BChE-Br; Lane 3: Marker; Lane 4: BChE-PDMAA-N_3; Lane 5: BChE-PDMAA-N_3 + Proteinase K; Lane 6: Proteinase K.

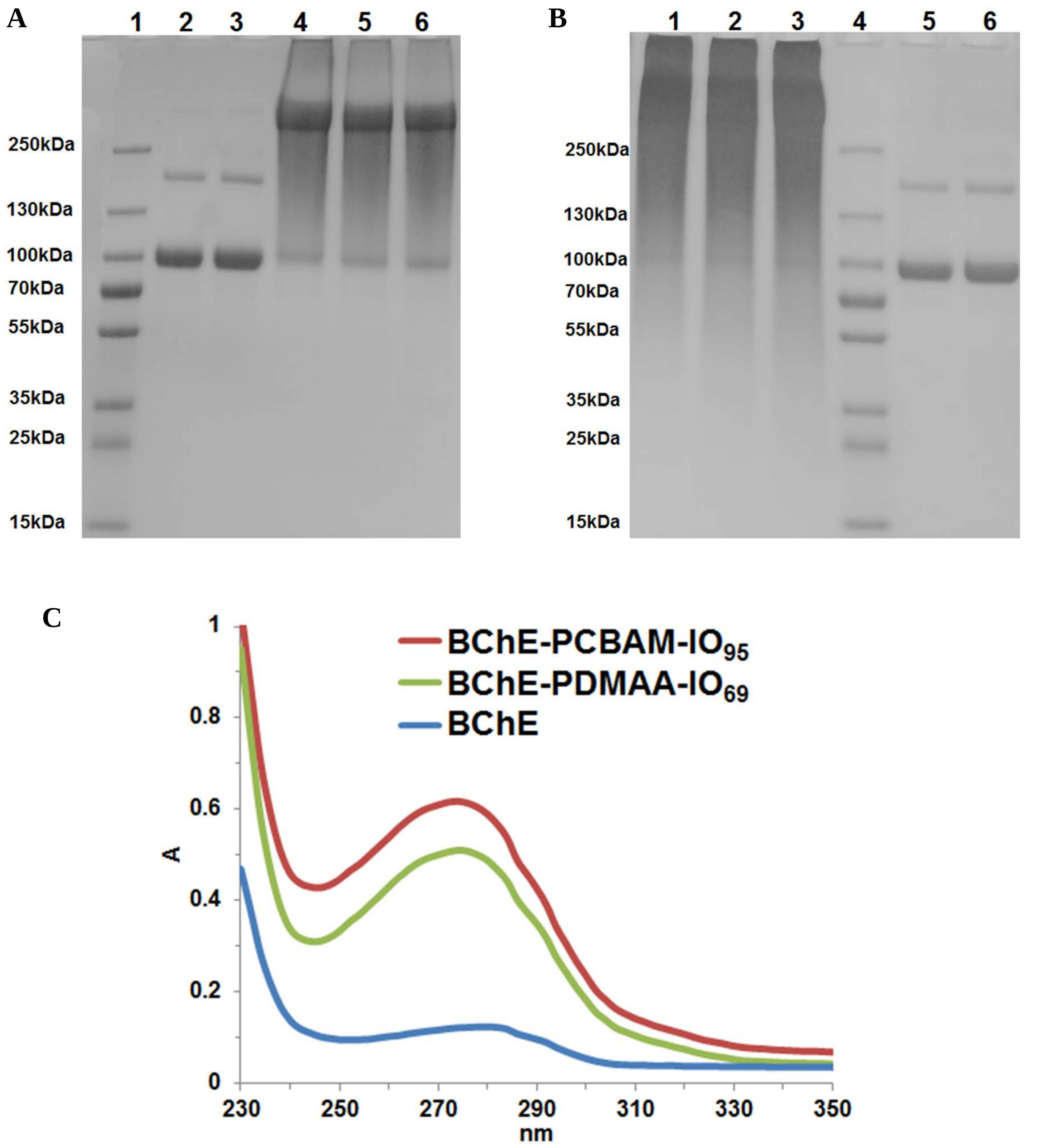

Figure S4. A) SDS-PAGE analysis of BChE-PDMAA-IO conjugates. Lane 1: Marker; Lane 2: BChE; Lane 3: BChE-Br; Lane 4: BChE-PDMAA-IO_{69}; Lane 5: BChE-PDMAA-IO_{44}; Lane 6: BChE-PDMAA-IO_{15}, B) SDS-PAGE analysis of BChE-PCBAM-IO conjugates. Lane 1: BChE-PCBAM-IO_8; Lane 2: BChE-PCBAM-IO_{49}; Lane 3: BChE-PCBAM-IO_{90}; Lane 4: Marker; Lane 5: BChE; Lane 6: BChE-Br. C) UV-VIS spectrum absorbance of BChE, BChE-PDMAA-$IO_{69,}$ and BChE-PCBAM-IO_{95} conjugates at 2 μM concentrations of BChE.

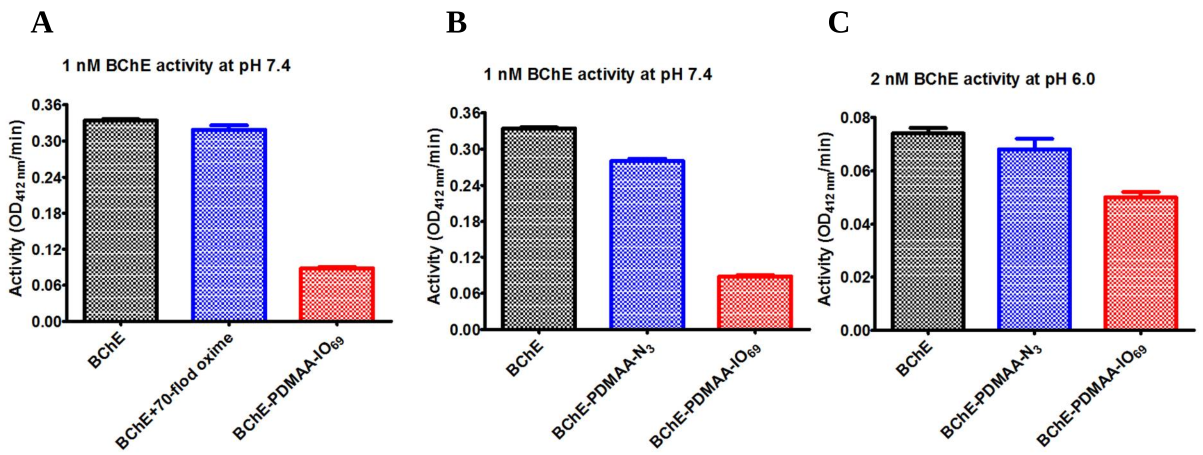

Figure S5. Enzymatic activities of BChE, BChE in the presence of 70-fold alkyne-imidazolium-oxime, BChE-PDMAA-$N_{3,}$ and BChE-PDMAA-IO_{69} conjugates at (A and B) pH 7.4 and (C) pH 6.0.

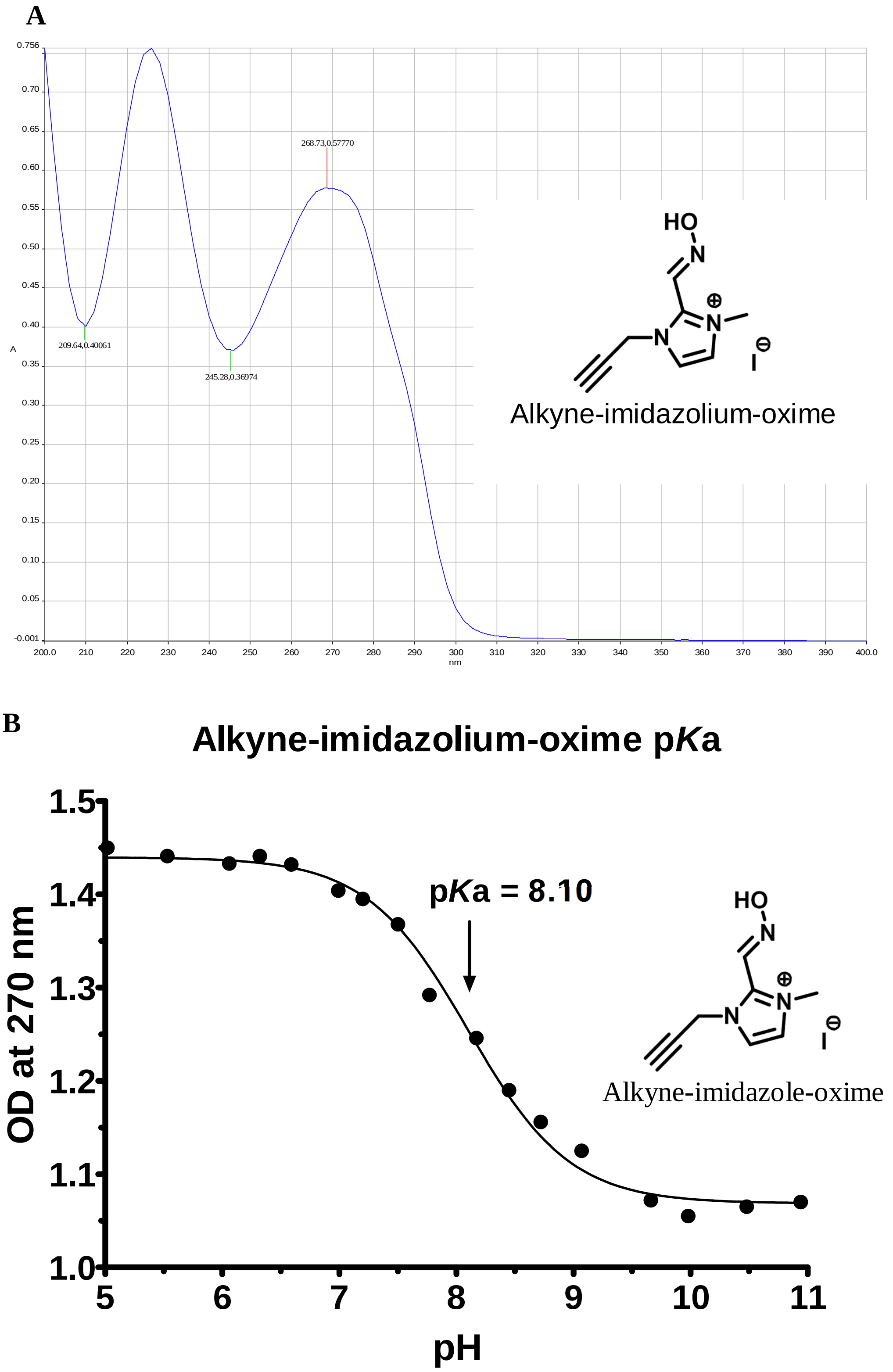

Figure S6. A) UV-VIS spectrum absorbance of Alkyne-Imidazolium-Oxime, B) p*K*a determination of alkyne-imidazole-oxime

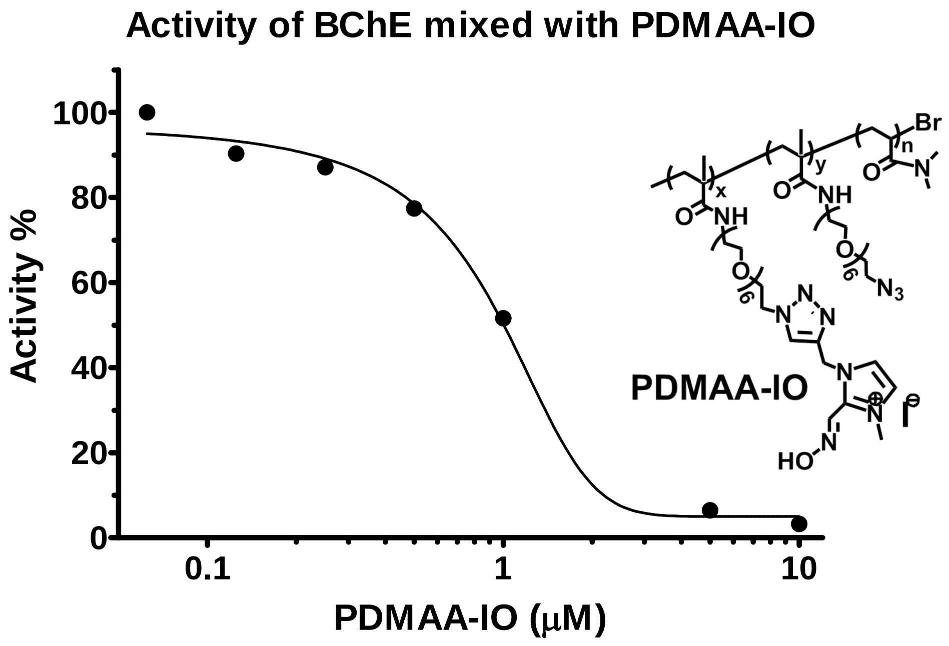

Figure S7. Activity assay of native BChE mixed with different concentrations of PDMAA-IO.

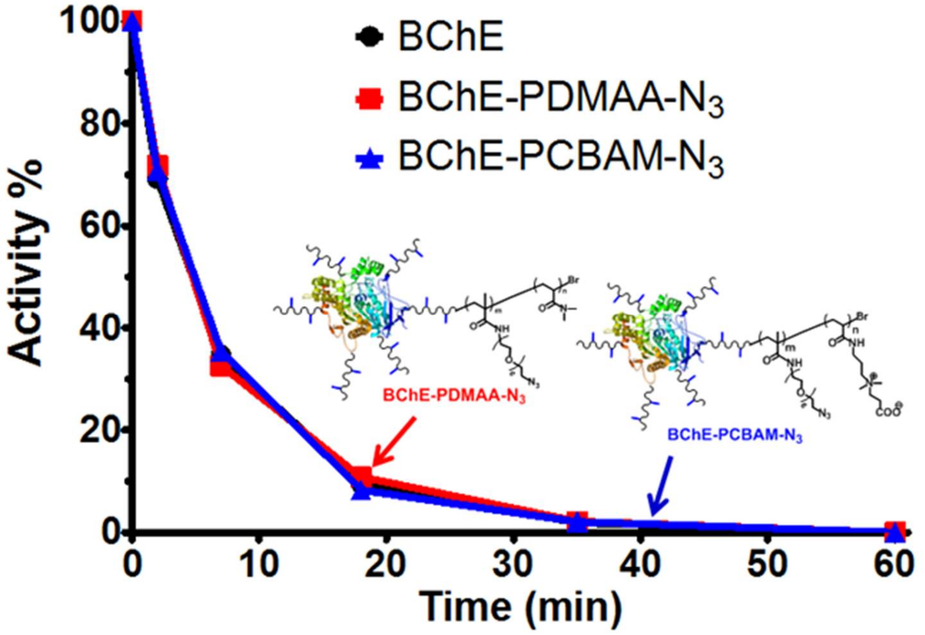

Figure S8. Inhibition assay of 20 nM (protein) BChE, BChE-PDMAA-N_3 and BChE-PCBAM-N_3 by 3.2-fold stoichiometric excess of POX at pH 7.4 for 60 min.

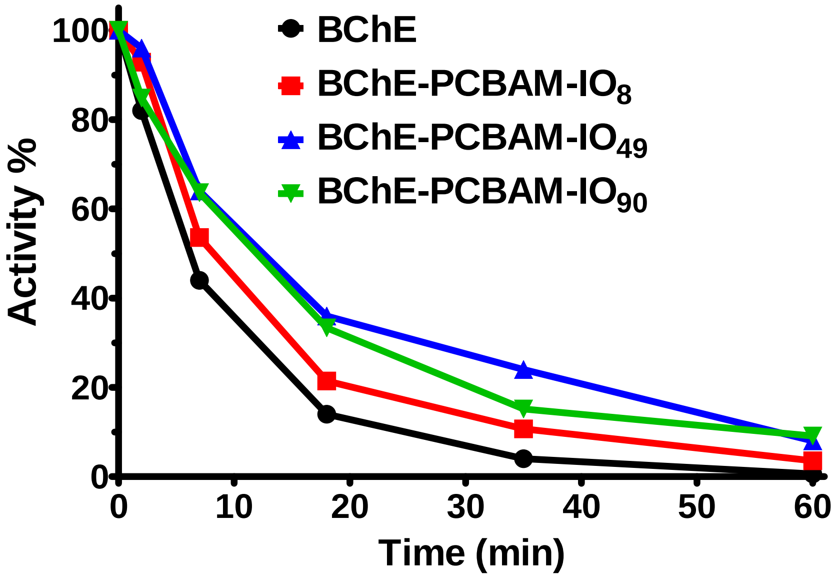

Figure S9. Inhibition assay of 20 nM (protein) BChE and BChE-PCBAM-IO conjugates by 3.2-fold stoichiometric excess of POX at pH 7.4 for 60 min.

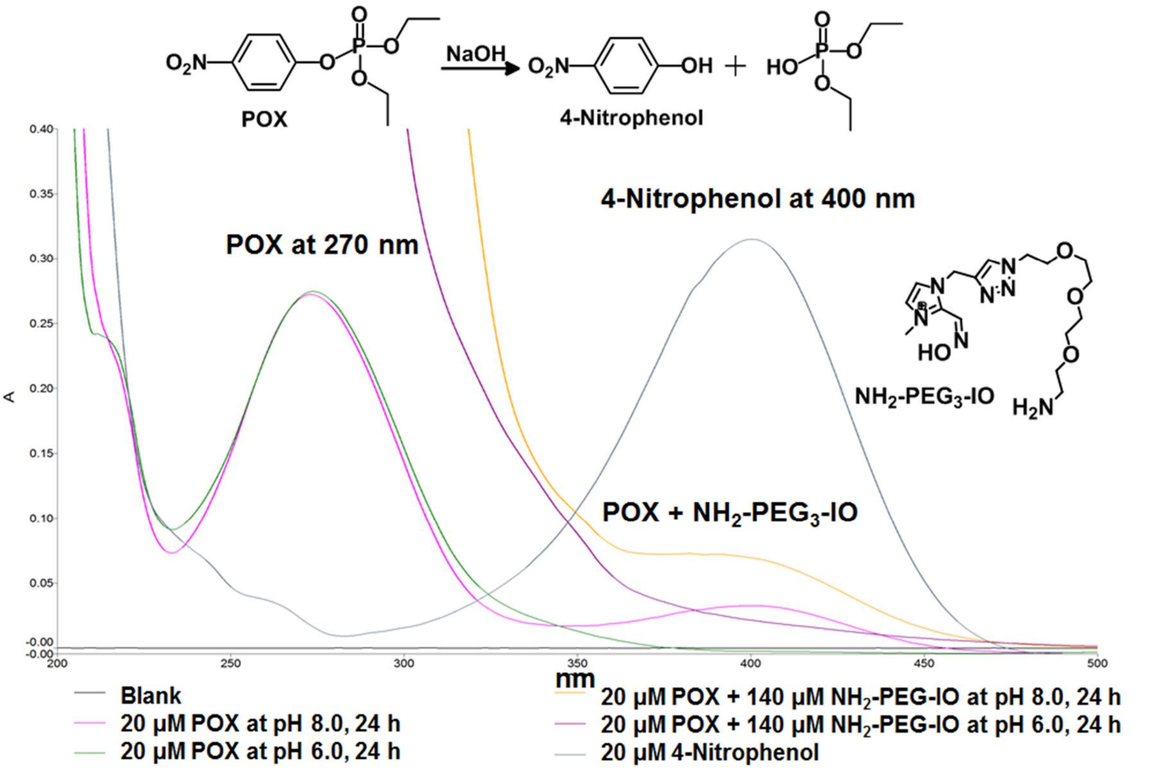

Figure S10. UV-VIS absorbance analysis of 20 µM POX mixed with 140 µM NH_2-PEG_3-IO at r.t. for 24 hours at pH 6.0 and 8.0.

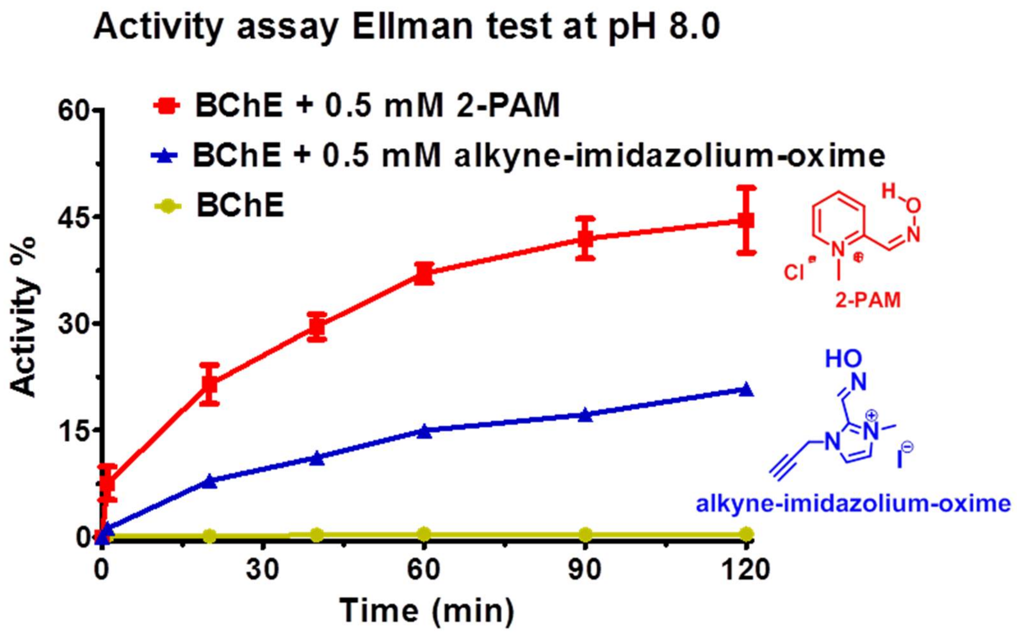

Figure S11. Reactivation assay of POX-inhibited native BChE by 0.5 mM of 2-PAM or alkyne-imidazolium-oxime. Results are presented as mean values ± standard deviation (n = 3).

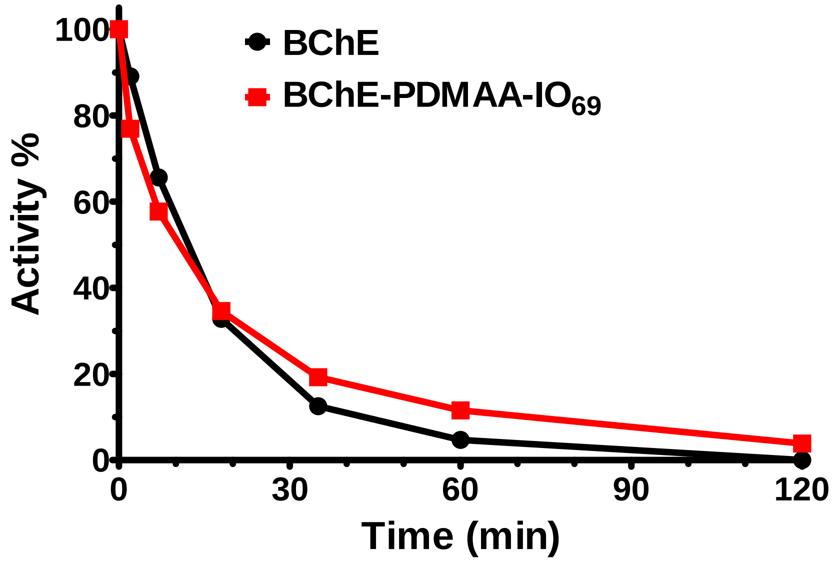

Figure S12. Inhibition assay of native BChE and BChE-PDMAA-IO_{69} (100 nM, protein) by 3.2-fold stoichiometric excess of POX at pH 6.0 for 120 min.

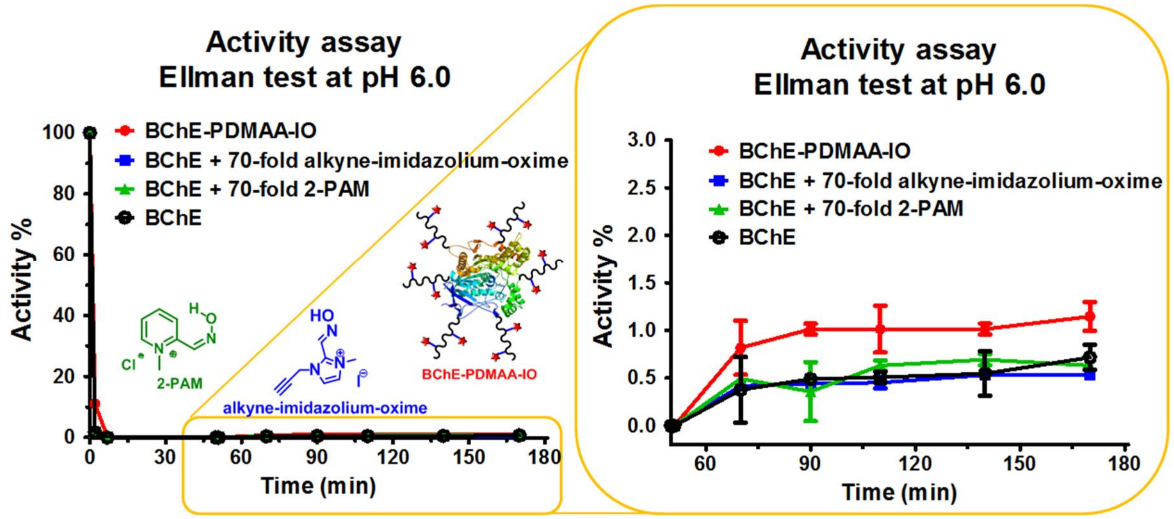

Figure S13. Reactivation assay of BChE and BChE-PDMAA-IO_{69} conjugate. 1 µM BChE-PDMAA-IO_{69} or BChE were inhibited by ten-fold excess of POX at pH 6.0 and then diluted 50-fold at pH 6.0. Results are presented as mean values ± standard deviation (n = 3).

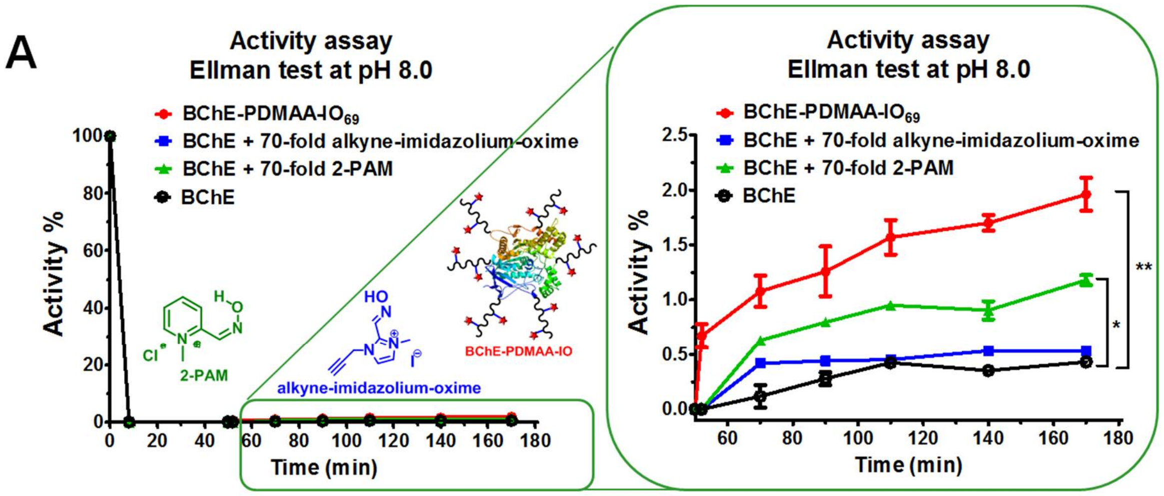

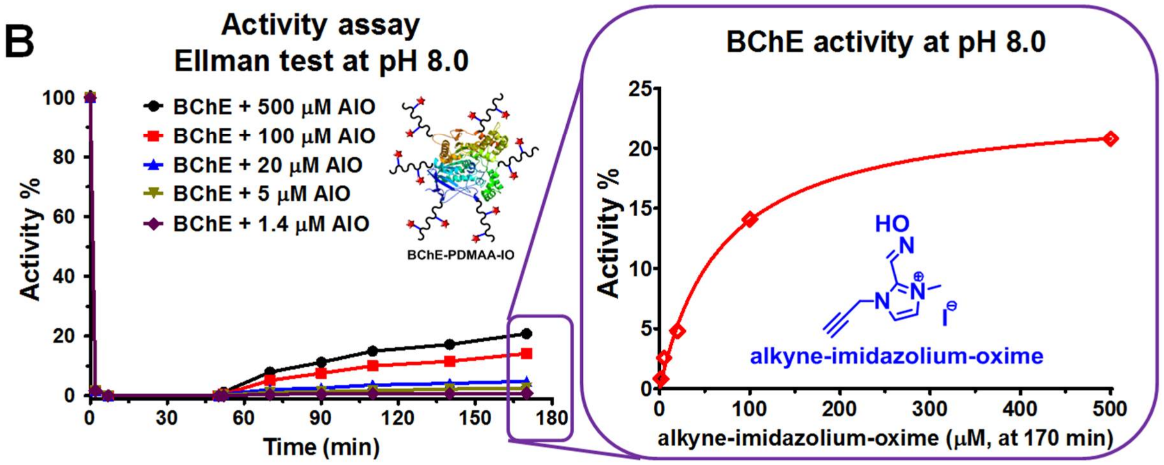

Figure S14. Reactivation assay of BChE and BChE-PDMAA-IO_{69} conjugate. (A)1 μM BChE-PDMAA-IO_{69} or BChE were inhibited by 10-fold excess of POX at pH 6.0 and then diluted 50-fold at pH 8.0. (B) 1 μM BChE were inhibited by 10-fold excess of POX at pH 6.0 and then reactivated at pH 8.0 by different concentrations of alkyne-imidazolium-oxime. Statistics (Student's *t*-test) was performed by comparing each treatment group with the corresponding native BChE group (*$p < 0.05$, **$p < 0.01$). Results are presented as mean values ± standard deviation ($n = 3$).

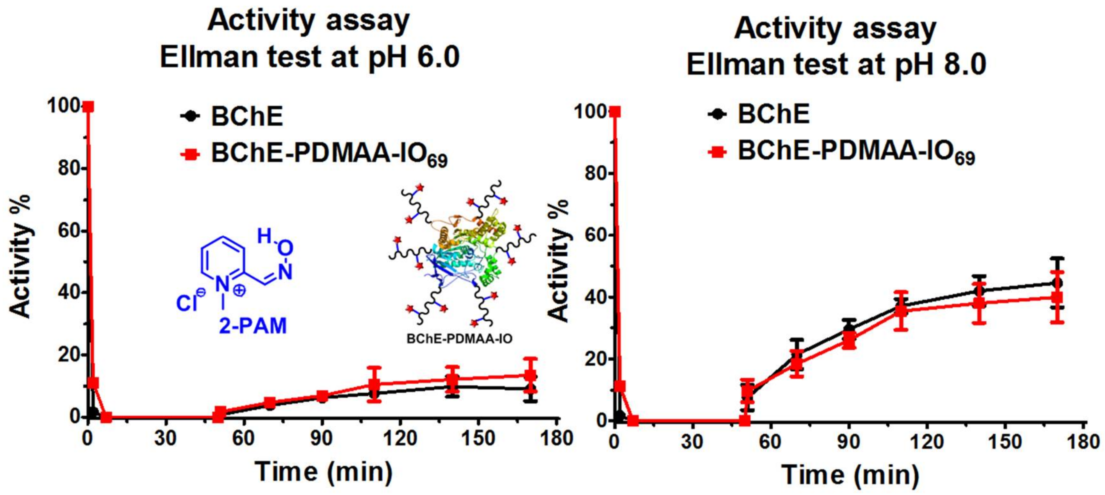

Figure S15. Reactivation assay of BChE and BChE-PDMAA-IO_{69} conjugate. 1 µM BChE-PDMAA-IO_{69} or BChE were inhibited by a 10-fold excess of POX at pH 6.0 and then diluted 50-fold at pH 6.0 (left) or pH 8.0 (right) with 0.5 mM 2-PAM.. Results are presented as mean values ± standard deviation (n = 3).

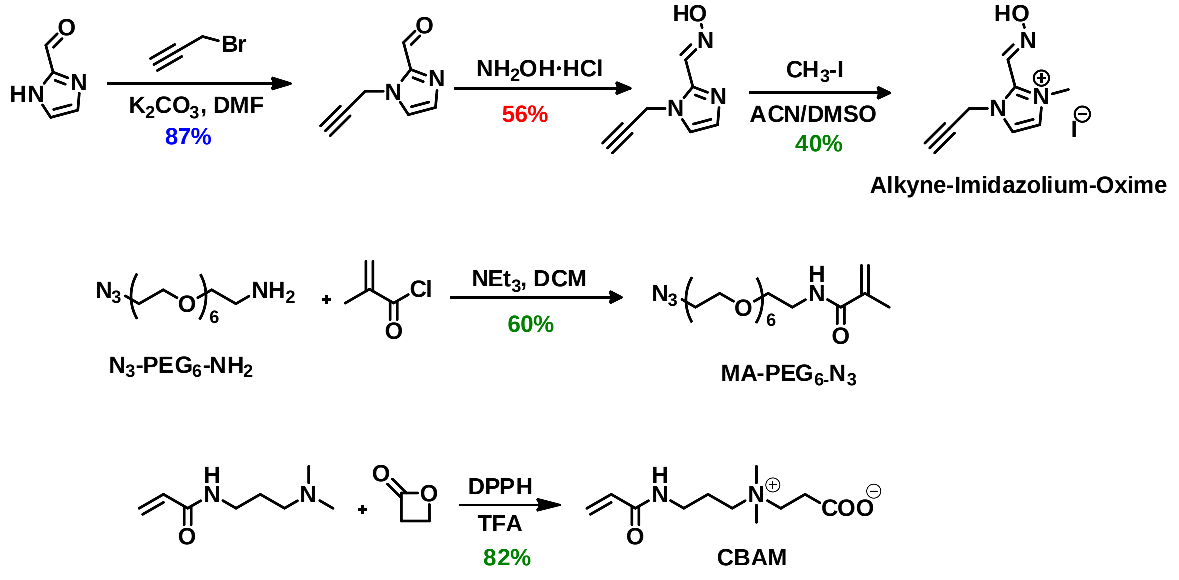

Figure S16. Synthesis of alkyne-imidazolium-oxime, MA-PEG$_6$-N$_3$ and CBAM

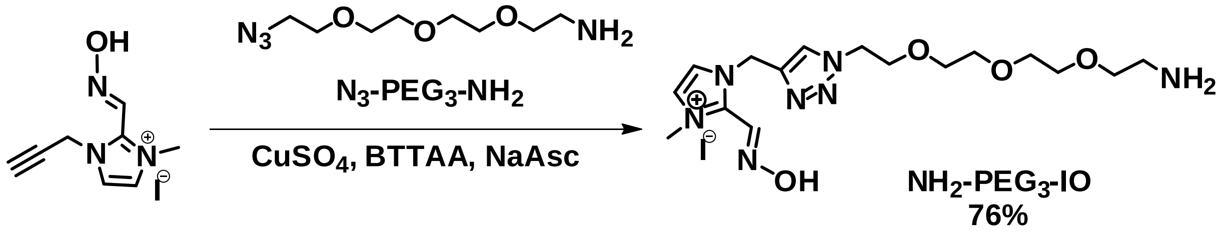

Figure S17. Synthesis of click reaction product NH$_2$-PEG$_3$-IO

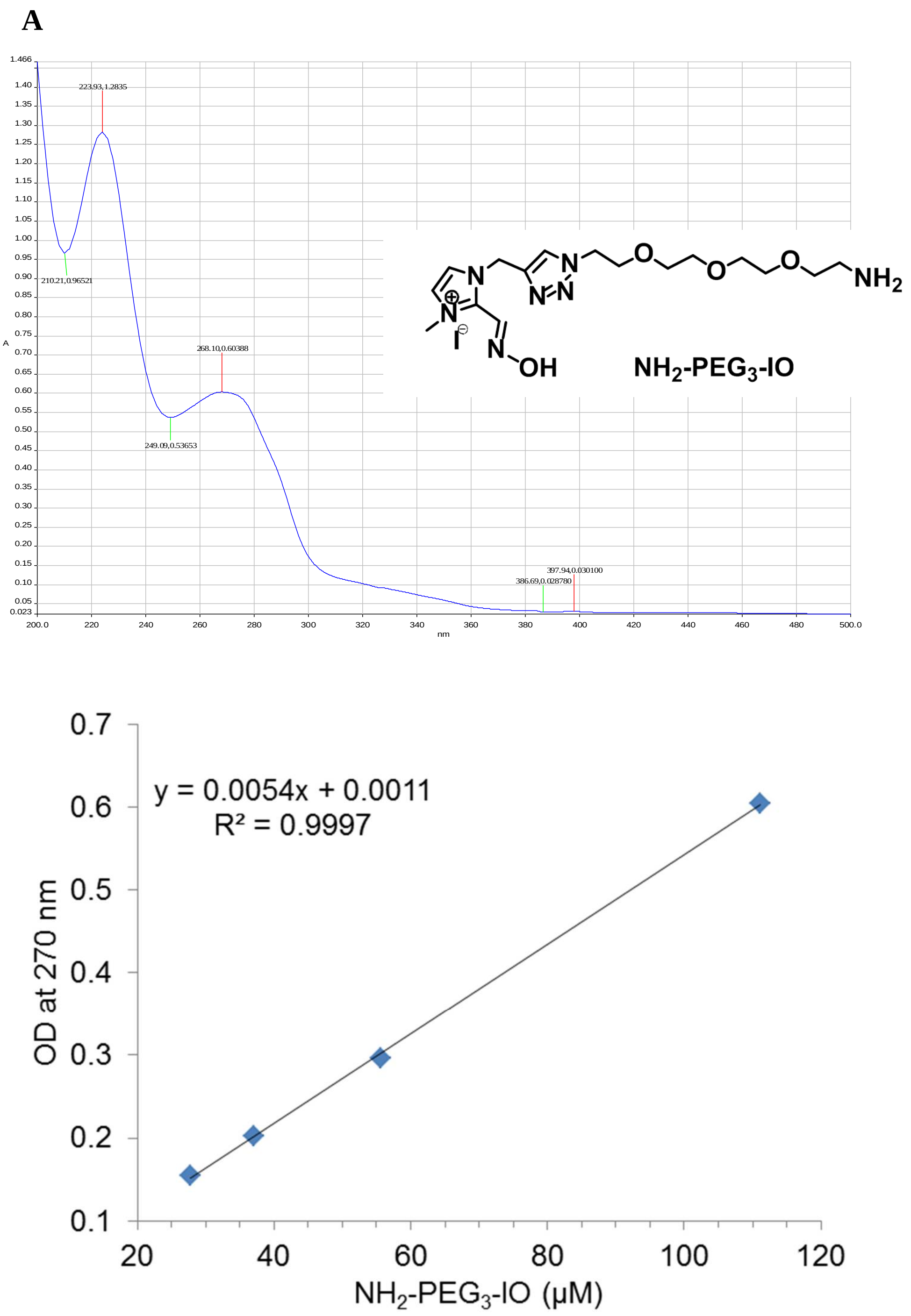

Figure S18. A) UV-VIS absorbance of NH_2-PEG_3-IO, B) standard working curve of NH_2-PEG_3-IO at 270 nm
